# Chemi-Inspired Silicon Allotropes—Experimentally Accessible Si_9_ Cages as Proposed Building Block for 1D Polymers, 2D Sheets, Single-Walled Nanotubes, and Nanoparticles

**DOI:** 10.3390/molecules27030822

**Published:** 2022-01-26

**Authors:** Laura-Alice Jantke, Antti J. Karttunen, Thomas F. Fässler

**Affiliations:** 1 Department of Chemistry, Technische Universität München Lichtenbergstr. 4, 85747 Garching, Germany; Laura.Jantke@lrz.tu-muenchen.de; 2 Department of Chemistry and Materials Science, Aalto University, 00076 Aalto, Finland

**Keywords:** silicon, allotropy, clusters, nanotubes, computational chemistry, density functional theory

## Abstract

Numerous studies on silicon allotropes with three-dimensional networks or as materials of lower dimensionality have been carried out in the past. Herein, allotropes of silicon, which are based on structures of experimentally accessible [Si_9_]^4−^ clusters known as stable anionic molecular species in neat solids and in solution, are predicted. Hypothetical oxidative coupling under the formation of covalent Si–Si bonds between the clusters leads to uncharged two-, one- and zero-dimensional silicon nanomaterials not suffering from dangling bonds. A large variety of structures are derived and investigated by quantum chemical calculations. Their relative energies are in the same range as experimentally known silicene, and some structures are even energetically more favorable than silicene. Significantly smaller relative energies are reached by the insertion of linkers in form of tetrahedrally connected Si atoms. A chessboard pattern built of Si_9_ clusters bridged by tetrahedrally connected Si atoms represents a two-dimensional silicon species with remarkably lower relative energy in comparison with silicene. We discuss the structural and electronic properties of the predicted silicon materials and their building block *nido*-[Si_9_]^4–^ based on density functional calculations. All considered structures are semiconductors. The band structures exclusively show bands of low dispersion, as is typical for covalent polymers.

## 1. Introduction

Silicon is the material of choice for trying to fulfill the energy demands for our steadily growing society [1]. Thus, many studies on silicon allotropes, as three-dimensional networks or materials of lower dimensionality, have arisen in the last couple of years. Silicon is indispensable for photovoltaics and other materials with semiconducting properties that act as devices of various kinds. However, α-Si, the thermodynamically most stable modification at ambient conditions with all Si atoms being sp^3^-hybridized, only has an indirect band gap. With the knowledge that the structure of a material is strongly linked to its properties, a suitable modification of the Si structure could end up in a thin layer material with a direct band gap.

The possibilities of obtaining new silicon-based materials are rather diverse, especially if such structures are predicted computationally. Direct band gap materials [2,3], materials with a quasi-direct band gap as well as other low-energy materials [3], have been introduced lately. Additionally, the fabrication of a two-dimensional monolayer of the lighter homologue carbon, graphene [4], and the elucidation of its optical and electronic properties raised the interest for two-dimensional structures of various kinds of elements [5,6,7,8,9]. In particular, the unique electronic situation for graphene gives rise to a vast variety of fascinating physical properties [10,11,12]. It was a short way along the periodic table to computationally search for analogous silicenes and other graphene-like element modifications [13,14,15,16,17].

**Figure 1:**
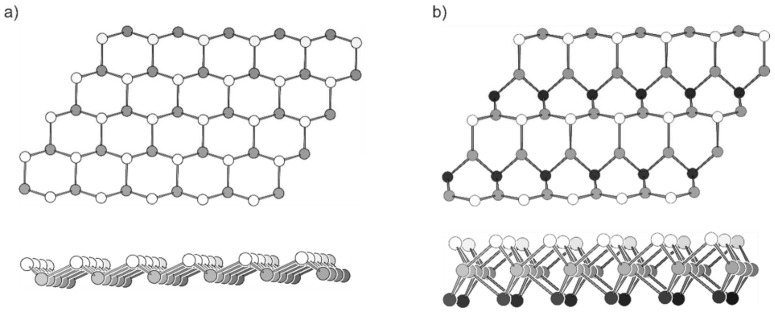
Top and side view of two-dimensional Si modifications as discussed in the literature. (**a**) The buckled honeycomb structure of silicene. (**b**) A sheet of two-dimensional Si in the MoS_2_ modification. The white, grey and black spheres represent Si atoms above, in and below the plane, respectively.

As an intriguing difference between silicon and carbon, Si does not tend to form double-bonds with sp^2^-hybridized Si atoms. Thus, the formation of a completely flat honeycomb net stabilized by its delocalized π-electrons is not possible in contrast to carbon. For Si, a sp^3^/sp^2^-hybridization takes place, and the honeycomb layer buckles instead of forming an aromatic flat sheet. The Si atoms that are both below and above the plane have an intrinsic Van der Waals interaction (Figure 1a) [14]. With the buckled structure, it could also be shown that the addition of Si atoms to pristine silicene lowers the total energy of the sheets, ending in dumbbell-like structures. Previous calculations have shown that the higher group 14 homologues prefer the dumbbell-like structures instead of the graphene-like honeycomb net, and the properties of their two-dimensional structures are also rather different from graphene [18,19,20].

Other 2D Si element modifications have also been computationally studied and suggested for example the MoS_2_-structure, which is less strained than silicene and has smaller relative energy (Figure 1b) [21]. However, no unsupported 2D Si modification could be synthesized yet.

Silicene has been first obtained experimentally and analyzed on a Ag(111) surface [22]. This first report was followed by other methods and substrates, which are well summarized in review articles by Kaloni et al. and Zhuang et al. [23,24]. A comprehensive overview of theoretical studies on this material is given in the review of Lew Yan Voon et al. [25]. The higher homologue germanene was first realized experimentally on an Au(111) surface [26].

Strongly connected to the discovery of graphene is the high interest in one-dimensional materials, such as nanotubes and -wires, also because they can be formed by a rolling up of graphene sheets, and they can even be made chiral dependent on the rolling axis. Such carbon nanotubes (CNTs) were first discovered in the early 1990s and inspired theoreticians to predict similar Si nanotubes (SiNTs) with comparable electronic and structural properties [27,28]. As it has also be seen for the flat silicene sheets, the predicted stable single-walled SiNTs are built of two types of Si atoms, which are either sp^3^- or sp^2^-hybridized and thus have puckered nanotubular structures [29]. Seifert et al. derived structural models involving either sp^2^ and sp^3^, or mixed sp^2^-sp^3^ hybridization, where the hypothetic sp^2^ single-walled SiNTs possess the best optical properties [30,31].

SiNTs are promising candidates for the next-generation materials of micro- and nano-electronic devices [32,33,34], as they have, for example, better performance with respect to electronic transport [35], and they are able to produce a current of about one order of magnitude higher than CNTs [36]. SiNTs have been obtained for the first time from thin solid Si films in 2001 and have directly been synthesized by chemical vapor deposition in 2002 [37,38], and later by molecular beam epitaxy on porous alumina and by self-organized growth of SiNTs with smaller diameter via hydrothermal synthesis [39,40,41].

Aside from nanotubes, also Si nanorods are important Si materials. They are compatible with the currently integrated circuit technology for bulk silicon, but with a diameter of 10 nm or less, quantum effects become important, and their properties vary significantly from that of bulk silicon [42]. Furthermore, they may to a higher extent conserve the sp^3^ hybridization, and they are experimentally accessible via various routes [43,44,45]. Recently, a one-dimensional Si nanorod with possible applications such as nontoxic LED material has been synthesized [46].

Finally, Si nanoparticles (Si-NP) became an emerging field, since in living organisms Si-NPs are metabolized to water-soluble orthosilic acid [47], allowing for biodegradation. Nano-sized carbon clusters such as fullerenes, which represent a whole family of hollow spheres and ellipsoids, as well as tubes, are known and readily accessible. On the basis of the *I_h_*--symmetrical buckminsterfullerene C_60_ [48], hollow cage molecules of the higher homologues Si_60_ and Ge_60_ have been investigated with DFT methods, but in contrast to carbon species they are strongly distorted. However, Si_60_ is predicted to be stable towards spontaneous disintegration up to 700 K [49]. The work of Yu et al. on intermediate-size silicon clusters includes annealing studies and starts from the bulk diamond lattice with 60 or 123 atoms [50]. Both result in cluster-aggregates with subunits like Si_6_, Si_7_, Si_10_, and Si_12_ cages looking very similar to the deltahedral Zintl clusters found for tetrel elements. If the sizes of the Si aggregates increase, and a spherical nanoparticle with a diameter of about 2.5 nm and 417 Si atoms is considered, the bulk remains in the α-modification, but the surface atoms reconstruct to minimize the number of dangling bonds [50]. Such Si-NPs have been dispersed among carbon fibers and used for lithium-ion batteries to combine the advantages of Si and C [51].

Recently, we introduced an alternative search strategy—a so-called chemi-inspired search—for the discovery of new silicon modifications with tailored properties based on the formation of well-defined materials with known and experimentally accessible building blocks, which we primarily applied for tetrahedral frameworks [52].

We now extend this idea to another class of compounds, which are also based on tailor-made building units. Deltahedral Zintl clusters of Group 14 elements have well-defined structures which are retained upon solvation and have the capability of oligo- and polymerization [53]. Such clusters have served as seed crystals for the synthesis of Si nanoparticles [54]: they are stable in solution, and have been structurally characterized as anions in the solid state [55].

Here, we investigate one- and two-dimensional Si nanostructures with intact Si_9_ clusters as building blocks, using quantum chemical methods (see Section 3). We found several new structures with relatively low energy: (a) three two-dimensional networks, in two of which Si_9_ units are connected by tetrahedrally bonded Si atoms; (b) four polymers with and without linker atoms between the Si_9_ units and (c) several tubes and spheres derived from slabs in which the Si_9_ clusters are directly linked. Some of the proposed structures are analogous to structures that have been found for related Ge-based materials [56]. The consideration of tetrahedrally coordinated Si linker atoms is, however, introduced for the first time, and leads to different structures with significantly reduced structural strain.

## 2. Results and Discussion

### 2.1. Reinvestigation of Silicene

For an adequate comparison of the stability of our new structures with that of the experimentally known silicene, we calculated the latter, resulting in a buckled honeycomb net with Si–Si bond lengths of 2.27 Å and a bond angle of 116.2° applying the same quantum chemical methods and basis sets (DFT-PBE0 method, see Section 3). The basic {^2^_∞_[Si]^−^ structure unit, according to a 1 × 1 unit cell resulting in a condensation of D_3d_ symmetric hexagonal rings as they occur in CaSi_2_, is chosen [57]. Such honeycomb silicene is experimentally realizable on metal surfaces and may one day be accessible by peeling it off of the surface [22,58,59,60,61]. It has been suggested that the deviation from a perfect structure would result in energetically more favorable free-standing 2D silicon sheets [25]. Such structures are, e.g., based on reconstructed $\sqrt{3}$ × $\sqrt{3}$, 5 × 5, or 7 × 7 supercells, and result in an energy lowering of about 0.05 eV/atom, with respect to perfect silicene [19]. From now on, the energies of the new Si species are discussed relative to silicene.

### 2.2. The Nido-[Si_9_]^4–^ Cage and Oligomeric Species

**Figure 2:**
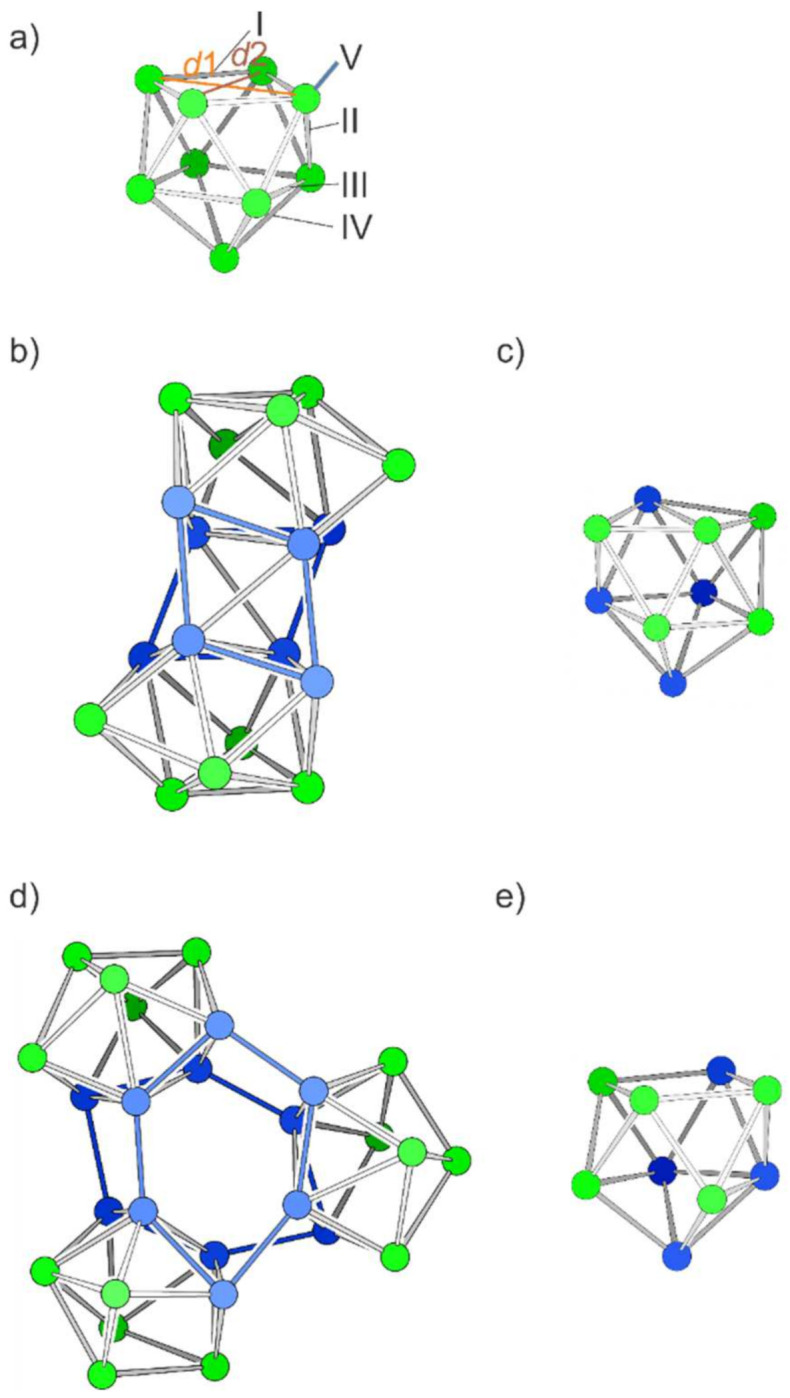
Structures of [Si_9_]^4–^ and neutral **S_2_**, (Si_9_)_2_ and **S_3_**, (Si_9_)_3_. (**a**) The optimized [Si_9_]^4–^ cluster. The labels refer to the distinguishable bond types in a C_4v_-symmetric polyhedron. Label V refers to *exo*-bonds from any vertex of the open square. (**b**) Dimer **S_2_**; the blue atoms refer to the eight atoms of the open square used for the fusion (newly formed bonds are shown in blue). (**c**) One nine-atomic unit of **S_2_**. (**d**) Trimer **S_3_**, obtained by connecting the atoms of the open squares and forming a central hexagonal prism shown with blue bonds. (**e**) A nine-atomic unit of **S_3_**. Si atoms with *exo*-bonds are shown in blue, the others in green.

In the following, all structures are derived by connecting *nido*-[Si_9_]^4–^ units. The basic unit corresponds to a monocapped square antiprism with an ideal *C*_4*v*_ point group symmetry (Figure 2a). In analogy to the hydroborane [B_9_H_9_]^4-^ the deltahedral [Si_9_]^4–^ structure can be understood as a 40-valence electron *nido*-structure [62,63,64]. The experimentally observed *nido*-Si_9_ cluster generally deviates slightly from this ideal symmetry. The deviation from *C*_4*v*_ symmetry is best described by the ratio of the diagonal lengths of the open square (*d*1/*d*2, Figure 1a), which is 1.00 for *C*_4*v*_ symmetrical clusters. The ratios for Si_9_ in solvate crystals are *d*1/*d*2 = 1.01–1.20 [65,66], and in solid A_12_Si_17_ [67], they are *d*1/*d*2 = 1.04–1.20 for K_12_Si_17_ and *d*1/*d*2 = 1.08–1.20 for Rb_12_Si_17_. Our optimized *nido*-[Si_9_]^4–^ shows a value of *d*1/*d*2 = 1.03, regardless of starting from a distorted or from a *C*_4*v*_ symmetrical input. Thus, many of the novel materials introduced in this work involve perfect *C*_4*v*_ symmetrical clusters as starting structures prior to geometry optimization. By contrast, for the related Ge_9_ clusters, perfectly *C*_4*v*_ symmetrical clusters are experimentally observed in the Zintl phase K_4_Ge_9_ [68], which is, however, not accessible for Si to date. Si_9_ clusters with approximate or almost perfect C_4v_ symmetry are found in binary phases and in the solution [55,67,69]. Population analyses of isostructural [Si_9_]^4–^ and [Ge_9_]^4–^ clusters show only minor differences (see Supplementary Materials). Such nine-atomic clusters can also be understood as super atoms with the magic number of 40 valence electrons [70,71], which would nevertheless rather crystallize as more spherical deltahedral cages as tricapped trigonal prisms in *D*_3*h*_ symmetry.

For deriving the novel, hypothetical structures the nido-clusters are connected at the vertices of the open square face of the monocapped quadratic antiprism, the cluster face whose atoms are usually involved in functionalization reactions [72,73,74]. Each *exo*-bond formation leads to a reduction in the negative charge (by formal oxidative coupling), and thus four *exo*-bonds per cluster result in a neutral species. Small neutral (Si_9_)_2_ (**S_2_**) and (Si_9_)_3_ oligomers (**S_3_**) are formed upon the connection of two and three *nido*-[Si_9_]^4–^clusters, respectively, where the novel bonds arise between atoms of the open square (bonds denoted by V in Figure 1a). Even though a direct connection between Si_9_ clusters has not been observed yet, the *exo*-bond formation between Ge_9_ clusters is well established [75,76,77,78,79,80].

The starting geometry for **S_2_** with *D*_4*h*_ symmetry corresponds to a cube formed by the parallel open faces which are each capped by two tilted opposing square pyramids. Relaxation of the structure leads to a real minimum (confirmed by frequency calculations) by reducing the symmetry to *D*_2*d*_ (Figure 2b). The basic Si_9_ unit rearranges during optimization to a minimum structure which corresponds to the connection of two triangular sides of the Si_9_ cluster including one more atom each (blue structure part in Figure 2b,c). A related arrangement of atoms has recently been observed in the (disordered) intermetalloid cluster {[CuSn_5_Sb_3_]^2–^}_2_ with a rather similar number of valence electrons per nine-atomic unit (38 valence electrons (VE) in contrast to 36 VE for **S_2_**) [81]. The relative energy of the minimum structure of Si_18_ is 0.16 eV per atom higher in comparison with that of silicene.

The connection of three Si_9_ clusters to the spherical oligomer (Si_9_)_3_ (**S_3_**) leads after relaxation to the *C*_1_-symmetric oligomer shown in Figure 2d with two puckered six-membered rings as a basic unit (blue in Figure 2e). Interestingly, the *nido*-type units are retained as distorted cages in both structures **S_2_** and **S_3_**, visualized in Figure 2d,e, respectively (Table 1). The relative energy of **S_3_** is about 0.05 eV lower than that of **S_2_**. Both oligomers, the one with 18 and the one with 27 atoms, show larger Si–Si distances than the monomers.

### 2.3. Spherical Nanoparticles

**Figure 3:**
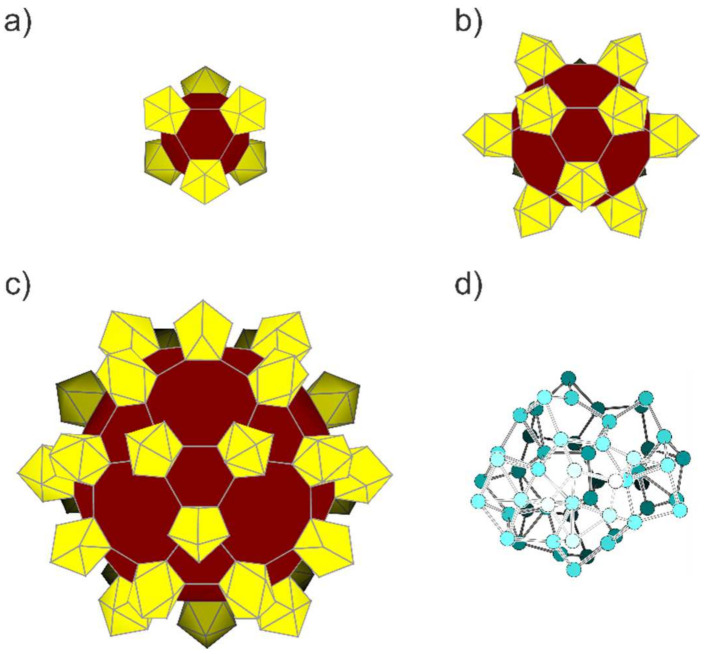
Nanosized spherical Si_9_ oligomers derived from nido-[Si_9_]^4–^ clusters directly connected with exo-bonds from all vertices of the open square. (**a**) **S_6_**, (Si_9_)_6_, consisting of 54 atoms. (**b**) **S_12_**, (Si_9_)_12_, consisting of 108 atoms. (**c**) **S_30_**, (Si_9_)_30_, consisting of 270 atoms. (**d**) A cutout from α-Si with 54 atoms.

Nanoparticles considered in this work are hollow spheres with six (**S_6_**), twelve (**S_12_**) and thirty Si_9_ units (**S_30_**). In contrast to the oligomers, these larger structures retain the *C*_4*v*_ symmetry of the basic Si_9_ clusters during relaxation. **S_6_** completes the faces between the clusters to irregular hexagons (Figure 3a). Together with the basal cluster squares, they form a truncated octahedron with *O_h_* symmetry which is identical to the one described in reference [55]. Compared with the bare *nido*-[Si_9_]^4−^ clusters, the interconnection results in a shortening of the Si-Si distances within the open square from 2.46 Å to 2.37 Å for the optimized cluster anion and a slight elongation of all other distances (Table 1). The *exo*-bonds of 2.30 Å become shorter than the *intra*-cluster bonds, which is in agreement with experimental findings for the dimers and polymers of the Ge_9_ clusters [75].

The central connecting unit in **S_12_** corresponds to a truncated cuboctahedron with *O_h_* point symmetry and the square faces being decorated with Si_9_ clusters (Figure 3b). The *exo*-bonds form octagons and hexagons with distances of 2.30 Å corresponding to a localized single bond, whereas the distances along the edges of the squares are 2.36 Å. The largest hollow sphere was optimized in *I_h_* symmetry leading to a truncated icosidodecahedron with Si–Si distances of only 2.25 Å for {Si_9_} *exo*-bonds and slightly shorter distances of 2.35 Å for the *intra*-cluster connections (Figure 3c). The relative energy is reduced to 0.05 eV for **S_6_** and further to 0.02 eV for **S_12_** and **S_30_**.

Although the nanoparticles have large surface to bulk ratios and do not correspond to bulk-like sections, the energy difference to related clusters that arise as 54-atomic cutouts of α-Si are rather small. The energy difference between the structure of **S_6_**, which also consists of 54 atoms, and the optimized structure of this cutout (shown in Figure 3d) is 0.07 eV/atom. Other approaches such as the compressing liquid method lead to other structures, in which the usual surface to bulk ratio of smaller particles raises their total energy [82].

### 2.4. Polymers

**Figure 4:**
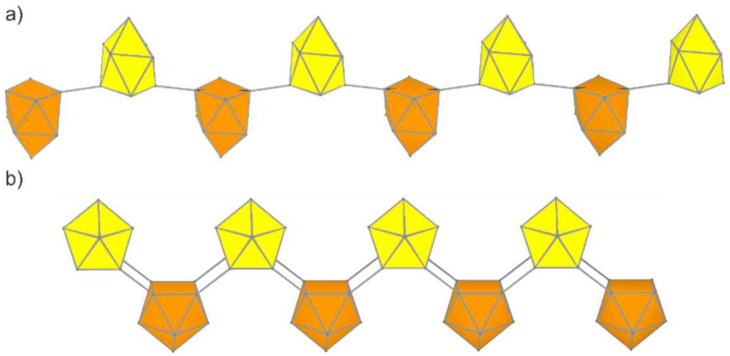
Predicted Si_9_ polymers built exclusively of [Si_9_] units. (**a**) Si9−Si9∞1n (**P1**). (**b**) Si9=Si9∞1n (**P2**) Clusters pointing up- and downwards are shown in yellow and orange, respectively.

A linear arrangement of interconnected Si_9_ clusters leads to polymers. Si9−Si9∞1n (**P1**) is composed of Si_9_ clusters connected with two center/two electron bonds via two opposing atoms of the open square. Its rod group is *p $\bar{1}$*, and the open face of the clusters are alternately aligned up and down. This neutral polymer can be regarded as the chemi-inspired structure that is formed upon oxidative coupling of the known [Si_9_]^2–^ clusters [52,83]. In contrast to the tricapped trigonal prismatic structure of the monomer, the two-fold linked clusters adopt a distorted *C*_2_ symmetric shape. After structure optimization, the connecting *exo*-bonds show a length of 2.32 Å, and the *intra*-cluster Si-Si distances range from 2.37 to 2.49 Å (Figure 4a). The relative energy with respect to silicene is Δ*E* = 0.21 eV per atom (which is Δ*E* = 1.02 eV relative to bulk α-Si), which is the highest value obtained in our calculations. The structure is not a true local minimum, as it shows an imaginary frequency of 98*i* cm^–1^. It could be identified as an asymmetric rotation of the clusters along the translational axis. We tried to distort the structure in the direction of the imaginary mode and re-optimize the geometry, but the optimization ended in the same structure, with the imaginary mode still present.

An alternative linear connection of Si_9_ units is realized in the neutral polymer Si9=Si9∞1n (**P2**, Figure 4b), in which the four atoms of the open square are pairwise connecting neighboring Si_9_ units. This structure is a novel 1D material that has not been considered for other tetrel elements. A comparable arrangement of tetrel clusters with rather long *exo*-bonds has been found in the anion [Pb_9_]^4-^ of the neat solid K_4_Pb_9_[67,84].

The optimized polymer **P2** has *pmm*2 rod group symmetry with slightly distorted *C*_4*v*_-symmetric clusters (d_1_/d_2_ = 1.01). The distances are marginally longer than in the nanoparticles, with 2.38 Å within the open square and 2.32 Å for the *exo*-bonds. The torsion angle between the open square and the square formed upon the polymerization is 141.6°; the relative energy of Δ*E* = 0.14 eV per atom is smaller than for **P1**.

Structures with units similar to **P1** of the larger homologue Ge show an anionic strand Ge9∞1n2− in which each cluster entity is two-fold negatively charged. In this experimentally accessible polymer, the deltahedral shape is retained [75], and the ratio of contacts follows the same trend as for **P1**. It has shorter *exo*-bonds (2.49 Å) and longer *intra*-cluster distances (2.55–2.84 Å). In the Ge oligomers [(Ge_9_)_3_]^6–^ and [(Ge_9_)_4_]^8–^ [76,77,78,79], as well as in the computationally derived double-linked polymer, stabilized by Rb cations as Rb_2_Ge_9_ [85], the Ge_9_ clusters are connected via pairs of atoms of the capped square. However, the Ge_9_ subunits show the same *C*_4*v*_-symmetric shape. A covalent connection of four deltahedral Ge9 clusters with nine Ge atoms, also comprising five-membered rings between the cluster units, is found in the largest known polyanion [Ge_45_]^12-^ [80].

### 2.5. Si Allotropes with Two-Dimensional Structure

Termination of the bulk such as the surface of a single crystal consequently results—especially for covalent compounds—in the reconstruction of the surface atoms. This is well known for the Si(100) surface atoms that build buckled, asymmetric surface dimers, along with a 2 × 1 reconstruction or a 7 × 7 reconstruction of the Si(111) surface [86].

This tendency becomes more pronounced, the thinner the layers become, and is rather significant, if the layer consists of only a single atom, similar to silicene. A major driving force for reconstruction is the reduction in unsaturated surface atoms with dangling bonds. The reduction in these dangling bonds will lower the total energy. Deltahedral Si_9_ clusters have the extraordinary characteristic of the framework being stabilized by forming delocalized chemical bonds in analogy to borane clusters that follow Wade’s rules based on the number of skeletal electron pairs [62,63], and they have a lone pair of electrons at each vertex atom. Thus, all Si atoms are “saturated” with respect to their valency towards the surface of the cluster.

**Figure 5:**
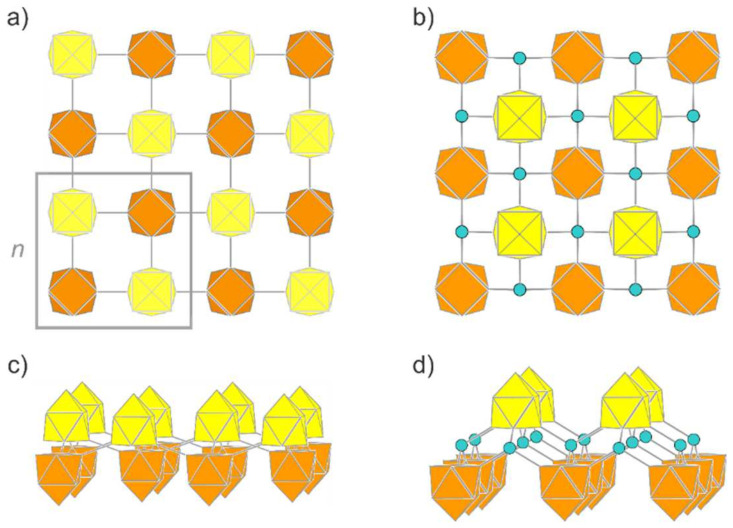
Novel 2D allotropes of silicon. (**a**) Top view of Si9∞2n (**L1**) consisting of directly connected Si_9_ units. The frame highlights the unit cell. (**b**) Side view of **L1**. (**c**) Top view of (Si92–Si2)∞2n (**L2**). The Si_9_ units are connected via sp^3^-hybridized Si atoms. (**d**) Side view of **L2**. Clusters pointing with the open face to alternating sides of the plane are shown in yellow and orange color, respectively. Bridging Si atoms are shown in turquoise.

In the case of Zintl clusters, we introduce building blocks which are known to persist in solution and as (charged or functionalized) nano-spheres. Thus, we introduce a Si9∞2n-layer (**L1**), which shows no tendency to reconstruct its surface, but converges to a true local minimum under retention of the starting geometry. The intact Si_9_ unit connects to four other Si_9_ clusters via all corners of the open square in classical 2c/2e bonds (Figure 5a,c), leading to an uncharged sheet. Chemically, each cluster undergoes four oxidative coupling reactions under reduction in the negative charges by one unit. The open square of the *nido*-type clusters alternately points up and down with respect to the plane connecting the clusters in one direction for polymer **P1**. Interestingly, in contrast to the one-dimensional polymers, the relaxation of this structure leads to *C*_4*v*_-symmetrical clusters and to more balanced *exo*- and *intra*-cluster bond lengths of 2.30 Å and 2.36 Å, respectively.

The analysis of the total energy reveals that **L1** is energetically equivalent to silicene (Table 1). This remarkable detail follows the observation that chemi-inspired structures tend to lead to local minima on the potential energy surface [52,83]. 

To reduce possible strain within the 2D network of **L1**, we introduced an additional tetrahedrally coordinated Si atom as a structural fragment, leading to (Si92–Si2)∞2n (**L2**). Silicon atoms that are covalently bonded to nine-atomic clusters are known for the higher homologue Ge, where the functionalization of Ge_9_ with silicon organyls leads to [Ge_9_(TMS)_3_]^-^ (TMS = Si[Si(CH_3_)_3_]_3_) [87,88,89], and also a crosslinking of these cluster units with different bridging atoms, for example the tetrel element Sn or the Group 12 elements Hg and Zn [90,91,92], and via organic bridges can be achieved [93].

**Figure 6:**
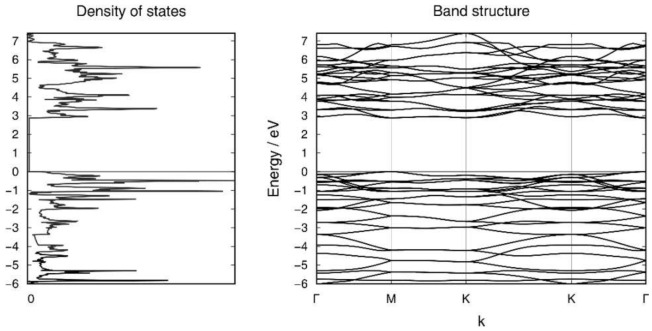
Electronic band structure and density of states of the layer structure **L2**.

The layer group of **L2** is *p*4/*nmm*, and the individual clusters are monocapped square antiprisms of perfect *C*_4*v*_ symmetry (Figure 5b,d). The distortion of the building block [Si_9_]^4–^ within **L2** is comparable to that within **L1**. The distance of the *exo*-bond to the tetrahedral Si atom is 2.33 Å with only small deviations (105.6° and 117.6°) from the tetrahedral angle, and thus rather close to the Si-Si bonding parameters in α-Si (2.36 Å and 109.4°). The relative energy of this novel allotrope amounts to −0.08 eV per atom with respect to silicene. The band structure and electronic density of states of **L2** are illustrated in Figure 6 and those of **L1** in Supplementary Materials. The (indirect) band gaps of **L1** and **L2** are 2.63 eV and 2.89 eV, respectively. The electronic bands of both structures show a rather low dispersion. In the case of L2, the band gap is very close to being direct instead of indirect. The Supplementary Materials also contain the structure (Si92–Si2)∞2non-top (**L3**), which bridges Si atoms with highly strained bond angles and thus has a higher relative energy than all the others discussed herein.

**Figure 7:**
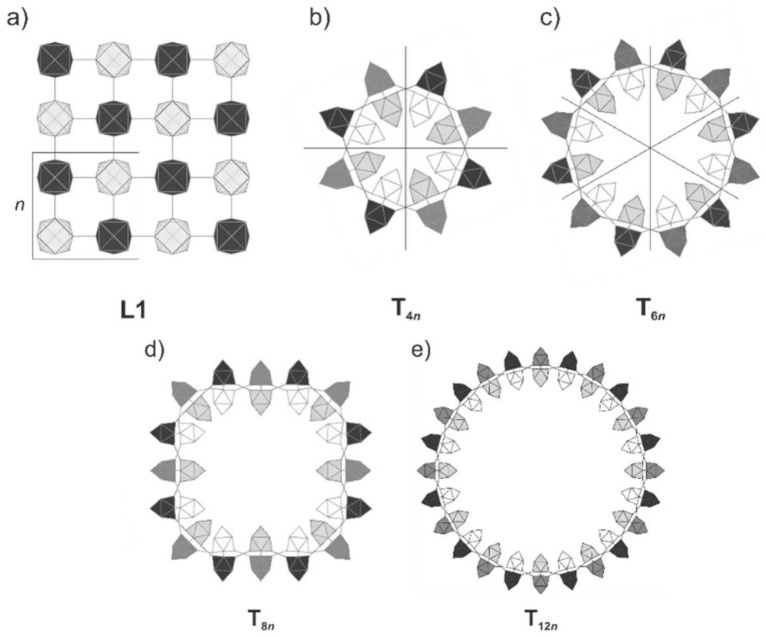
Tubes derived from rolling *m* units *n* of **L1**. (**a**) 2 × 2 section of **L1**; the unit cell is highlighted with a box. (**b**) **T_4*n*_** connecting a 2D sheet with 4-unit cells. (**c**) **T_6*n*_** connecting a 2D sheet with 6-unit cells. (**d**) **T_8*n*_** connecting a 2D sheet with 8-unit cells. (**e**) **T_12*n*_** connecting a 2D sheet with 12-unit cells.

The structure **L1** is very flexible, so that it can be rolled up to build closed tubes without a significant change in the relative energy. We modeled tubes with an inner diameter between 5.30 Å and 33.78 Å, realized by rolling **L1** to tubes **T*_mn_*** with ends joining after *m* (*m* = 4, 6, 8, 12) units *n* (Figure 7). Bond lengths, band gaps, total energy values (Table S1) and a more detailed discussion are given in the Supplementary Materials.

## 3. Materials and Methods

The structures of the considered silicon modifications were either optimized, starting from the structural parameters given in [56] or derived manually in an atom-by-atom manner or with a MATLAB script [94].

Silicene was recalculated based on a 1 × 1 unit cell of the Si sheet in CaSi_2_ [57]. It was optimized in the layer group *p $\bar{3}$* *m*1 (plane group no. 72), resulting in a buckled honeycomb net with *D*_3*d*_ point symmetry for the single six-membered rings.

The lattice and atomic positions of all structures were allowed to relax during the structural optimization within the constraints given by the layer, rod, or point group symmetry. All quantum chemical calculations for the solid-state species were carried out using the CRYSTAL program package with a hybrid DFT functional after Perdew, Burke, and Ernzerhof (DFT-PBE0) [95,96,97]. For silicon, a modified split-valence + polarization (SVP) basis set was applied [98]. The shrinking factor (SHRINK) for generating the Monkhorst-Pack-type grid of *k* points in the reciprocal space was set to 8 for all systems, resulting in 5 to 15 *k*-points in the irreducible Brillouin zone. For the evaluation of the Coulomb and exchange integrals, tight tolerance factors (TOLINTEG) of 8, 8, 8, 8, 16 were chosen. Default optimization convergence thresholds and an extra-large integration grid (XLGRID) for the density-functional part were applied in all calculations. Harmonic vibrational frequencies were calculated numerically to confirm the stationary point on the potential energy surface as a true minimum.

The investigations on the building blocks *nido*-[Si_9_]^4–^ and *nido*-[Ge_9_]^4–^ were carried out with the DFT-PBE0 hybrid DFT functional and def2-TVZP level basis sets for both elements Si and Ge using the Gaussian09 program package [99,100]. For compensation of the negative charge, a solvation model (polarizable continuum model, PCM) was applied [101]. For structure optimizations, very tight optimization convergence criteria (Opt = VeryTight) were combined with an ultrafine DFT integration grid. The systems were allowed to relax without symmetry restrictions. Harmonic vibrational frequencies were calculated analytically to confirm the stationary point on the potential energy surface as a true minimum.

The structural parameters for all optimized structures are listed in the Supplementary Materials.

## 4. Conclusions

We investigated Si allotropes based on the *nido*-[Si_9_]^4–^ cluster as a common building block. The relative energies of the considered structures are comparable to silicene which has been experimentally realized on metal substrates. The structure search is based on experimentally accessible and thus chemically relevant monomers—the building block *nido*-[Si_9_]^4–^, which is known to form oligomers and polymers via oxidation. The anionic molecular clusters have Si atoms at the convex surface of the clusters that avoids dangling bonds since all atoms are involved in the delocalized electron system of the deltahedral cluster.

A combination of these nine-atomic clusters to small uncharged (neutral) Si cluster units results in local energetic minima with a significant deformation of the originally *C*_4*v*_-symmetric subunits. The resulting relative energy is the highest for all structures based on *nido*-[Si_9_]. By contrast, the *C*_4*v*_ symmetry of the clusters is retained, when they become part of spherical hollow nanoparticles with diameters between 1.4 nm and 2.5 nm in and highly symmetrical shapes in which six, twelve or thirty {Si_9_} units are connected. The latter two have an identical relative energy of only 0.02 eV per atom, with respect to silicene.

The connection of *nido*-[Si_9_]^4–^ clusters to two-dimensional structures results in a sheet of directly connected clusters with a relative energy identical to that of silicene. If the clusters are bridged via sp^3^-Si atoms, and thus a fragment of α-Si is included, the relative energy can even be lowered to Δ*E* = −0.08 eV per atom, showing that the combination of fragments of stable structures lowers the total energy. Such a combination is performed twice in **L2**, as both the [Si_9_] clusters as well as tetrahedrally connected Si atoms are structural motifs known to be favored by Si. Consequently, **L2** is the structure with the lowest energy of all, and at the same time it is the most chemi-inspired one.

We conclude that a sheet built of Si_9_ clusters connected via tetrahedrally coordinated sp^3^-hybridized Si atoms is likely to be synthesized as a metastable allotrope—at least on metal surfaces, as it has been carried out in the case of silicene. As the *nido*-[Si_9_]^4–^ clusters do not suffer from surface problems such as dangling bonds and are stable in solution, the chemi-inspired layers derived from them may even be accessible without support. However, stabilization of the layers on metal substrates is also a prospective direction for experiments. Further computational studies with finite-temperature molecular dynamics are also expected to shed light on the dynamics of the free-standing and metal-supported layers.

Experimental studies have identified the stepwise transformation of the Zintl phase K_12_Si_17_ into solutions that contain solely *nido*-[Si_9_]^4–^ clusters. At a first step, the terminating of the Si_9_ anions by hydrogen have also been experimentally realized in line with hydrogen-terminated Si semiconductor nanostructures used in various models determined by first-principle calculations [69,102,103]. Although dangling bonds of terminal Si atoms are usually saturated by hydrogen atoms or by multiple bond formation, these clusters show that simultaneously with H termination, free electron pairs also avoid the formation of dangling bonds, thus enhancing the stability of silicon atoms in low oxidation states. Furthermore, recently we have experimentally shown that silicon atoms covalently bind to nine-atomic silicon clusters. Functionalization of Si_9_ with silicon organyls leads to [Si_9_(TMS)_2_]^2−^ and [Si_9_(TMS)_3_]^−^ (TMS= Si[Si(CH_3_)_3_]_3_) and comprise 17 and 21 directly connected Si atoms, respectively. These clusters are stable and can be characterized by ^1^H- and ^29^Si-NMR, as well as electrospray mass spectroscopic methods in solution. Several structures have been determined in the solid state by single-crystal X- ray diffraction methods [104,105], thus offering promising starting points for further studies in their controlled assembly into nanostructures

## Figures and Tables

**Table 1:** Summary of all considered chemi-inspired Si allotropes. The symmetry is given both for the whole structure and for individual {Si_9_} clusters within the structures. For the two-dimensional modifications derived from **L1**, two kinds of clusters are distinguishable, the inner (i) and the outer cluster (o). For distances to other clusters, a differentiation is made between bonds perpendicular (┴) and parallel (║) to the tube direction.

No.	Formula	Symmetry (of {Si_9_})	Bond Analysis (See Figure 2 for Labels)/Å							∆*E*/eV per Atom ^(3)^	Band Gap/eV
			I	II	III	IV	V	*d*1/*d*2	diameter/Å		
	Honeycomb sheet										
	Silicene	*C*_1_	-	-	-	-	2.27	-	-	0.00	1.32
	α-modification										
	Si_54_ nanocluster ^(4)^	*C*_1_	-	-	-	-	2.32–2.80	-	11.61	−0.02	1.52 ^(2)^
	Cluster unit and spherical oligomers										
	[Si_9_]^4− (1)^	(*C*_1_)	2.46	2.44 ^(5)^	2.63 ^(5)^	2.43	-	1.03	3.97		3.42 ^(2)^
**S_2_**	(Si_9_)_2_	*D*_2*d*_ (*C*_2*v*_)	2.52	2.50 ^(5)^	3.05 ^(5)^	2.47 ^(5)^	2.54 ^(5)^	1.54	7.74	0.16	3.14 ^(2)^
**S_3_**	(Si_9_)_3_	*C*_1_ (*C*_2*v*_)	2.45 ^(5)^	2.46 ^(5)^	3.04 ^(5)^	2.52 ^(5)^	2.40 ^(5)^	1.59	9.21	0.11	3.28 ^(2)^
	Nanoparticles										
**S_6_**	(Si_9_)_6_	*O_h_* (*C*_4*v*_)	2.37	2.46	2.79	2.51	2.30	1.00	13.72	0.05	3.31 ^(2)^
**S_12_**	(Si_9_)_12_	*O_h_* (*C*_4*v*_)	2.36	2.48	2.72	2.50	2.30	1.00	17.59	0.02	3.22 ^(2)^
**S_30_**	(Si_9_)_30_^(4)^	*I_h_* (*C*_4*v*_)	2.35	2.56	2.78	2.56	2.25	1.00	24.76	0.02	3.54 ^(2)^
	Polymers										
**P1^(6)^**	Si9–[Si9]∞1n	$p\bar{1}$(*C*_2_)	2.43 ^(5)^	2.56 ^(5)^	2.52 ^(5)^	2.57 ^(5)^	2.31	1.20	-	0.21	2.09
**P2**	Si9=[Si9]∞1n	*pmm*2 (*C*_2*v*_)	2.38 ^(5)^	2.46 ^(5)^	2.78	2.52 ^(5)^	2.32	1.01	-	0.14	2.45
	Two-dimensional modifications										
**L1**	Si9∞2n	$p{\bar{4}}_{2}m$(*C*_4*v*_)	2.36	2.49	2.68	2.50	2.30	1.00	-	0.00	2.63
**L2**	(Si92–Si2)∞2n	*p*4/*nmm* (*C*_4*v*_)	2.36	2.47	2.73	2.50	2.33	1.00	-	−0.08	2.89

^(1)^ The [Si_9_]^4−^ cluster is calculated with the Gaussian09 program package on a DFT-PBE0/def2-TZVP/PCM level of theory without any symmetry restrictions. ^(2)^ HOMO-LUMO gap. ^(3)^ The relative energy is based on Equation (1) (see above). ^(4)^ Frequency calculation could not be performed. ^(5)^ These values are averaged. ^(6)^ Not a true local minimum (see text).

## Data Availability

Not applicable.

## Supplementary Materials

Table S1: Structural and electronic properties of tubes derived from L1. Figure S1: Tubes derived from rolling m units n of L1. Table S2: Structural analysis of L3. Figure S2: Schematic representation of layer L3. Table S3: Partial atomic charges from natural population analysis (NPA) and Hirshfeld analysis. Figure S3: Band Structures and DOSs of polymers P1 and P2. Figure S4: Band Structures and DOSs of layers L1–L3. Text S1: Structural data of the studied structures.

## Sample Availability

Samples of the compounds are not available from the authors.
