# Supplementary material for: Chemi-Inspired Silicon Allotropes—Experimentally Accessible Si9 Cages as Proposed Building Block for 1D Polymers, 2D Sheets, Single-Walled Nanotubes, and Nanoparticles
Source: Molecules. 2022 Jan 26;27(3):822. doi: 10.3390/molecules27030822 (PMC8838638; doi:10.3390/molecules27030822)
Supplement: Supplementary file 1 [file molecules-27-00822-s001.zip › molecules-1545441-supplementary.pdf]

Supplementary Materials for

Chemi-inspired silicon allotropes – experimentally accessible Si_9 cages as proposed building block for 1D polymers, 2D sheets, single-walled nanotubes, and nanoparticles

Laura-Alice Jantke, Antti J. Karttunen, Thomas F. Fässler

Contents of the Supplementary Materials

1. Tubular one-dimensional modifications derived from $\{^2_\infty[Si_9]_n\}$ (L1)
2. Layer with $\{Si_9\}$ clusters on top of each other connected via sp^3-Si linkers
3. Population analyses for the basic $[Si_9]^{4-}$ unit in comparison to $[Ge_9]^{4-}$
4. Band structures and density of state (DOS) maps for considered one- and two-dimensional Si modifications
5. Structural data of the studied structures

1. Tubular one-dimensional modifications derived from $\{{}^{2}_{\infty}[\mathbf{Si_9}]_{\boldsymbol{n}}\}$ (L1)

Sheet **L1** is rolled up to tubes, just like reported for Ge_9 before.[1] The rolling bases on the same unit cell as the layer itself, and **L1** is rolled up to tubes **T** that close after ***m*** units ***n*** (compare Figure S1a). The smallest considered tube $\{{}^{1}_{\infty}[Si_9]_{4n}\}$ ($\mathbf{T}_{4n}$) completes a ring after eight Si_9 clusters pointing to the inside and outside, respectively. It is the smallest diameter permitting the inner clusters to co-exist without coordinating each other (Figure S1b). This small tube shows a relative energy of ΔE = 0.00 eV per atom identical to the relative energy of the sheet itself. Thus, rolling puts no significant additional strain to the structure, even though the diameter is quite small (5.30 Å and 21.66 Å for the inner and outer diameter, respectively, Table S1). Structurally, the clusters stay more or less equal to the ones in **L1**, but the *inter*-cluster distances are slightly enlarged to 2.31 Å and 2.32 Å along and perpendicular to the translational direction of the tube, respectively (Table S1).

The largest tube $\{{}^{1}_{\infty}[Si_9]_{6n}\}$ ($\mathbf{T}_{6n}$) is closed after 12 clusters (Figure S1e). It shows a slightly higher relative energy of 0.01 eV per atom, which is identical to the finding for Ge (structural parameters are in Table S1).

Two further nanotubes were studied, namely $\{{}^{1}_{\infty}[Si_9]_{8n}\}$ (T_{8n}, Figure S1c) and $\{{}^{1}_{\infty}[Si_9]_{12n}\}$ ($\mathbf{T}_{12n}$, Figure S1d) with relative energies in the range of the other allotropes derived from **L1** (Table S1). Unlike for Ge, for Si $\mathbf{T}_{8n}$ represents a stable modification with a tube with a square-shaped cross-section.

Table S1. Structural and electronic properties of tubes derived from L1 (compare Figure S1 and main text).

No.	formula	symmetry (of $\{Si_9\}$)	bond analysis (compare Figure S1)/Å I	II	III	IV	V	$d1/d2$	diameter/Å	Δ*E*/eV per atom	band gap/eV
$\mathbf{T_{4\mathit{n}}}$	$\{^1_\infty[Si_9]_{4n}\}$	*p*4*mm*	2.37i, 2.36^o	2.51^i, 2.48^o	2.66^i, 2.72^o	2.49^i, 2.50^o	2.31$^{\perp}$, 2.32$^{\parallel}$	1.11^i, 1.04^o	5.30^i, 21.66^o	0.00	0.87
$\mathbf{T_{6\mathit{n}}}$	$\{^1_\infty[Si_9]_{6n}\}$	*p*6*mm*	2.36^i, 2.37^o	2.50^i, 2.48^o	2.65^i, 2.69^o	2.49^i, 2.50^o	2.31$^{\perp}$, 2.32$^{\parallel}$	1.07^i, 1.03^o	12.66^i, 28.88^o	0.01	1.84
$\mathbf{T_{8\mathit{n}}}$	$\{^1_\infty[Si_9]_{8n}\}$	*p*4*mm*	2.36^i, 2.36o	2.50^i, 2.48o	2.67i, 2.70o	2.49i, 2.50o	2.31$^{\parallel}$, 2.31$^{\perp}$	1.05^i, 1.03^o	16.48^i, 37.25^o	0.00	1.26
$\mathbf{T_{12\mathit{n}}}$	$\{^1_\infty[Si_9]_{12n}\}$	*p*6*mm*	2.36i, 2.36o	2.49i, 2.49o	2.66i, 2.69o	2.49i, 2.50o	2.31$^{\parallel}$, 2.31$^{\perp}$	1.03i, 1.02o	33.78i, 50.17o	0.01	2.39

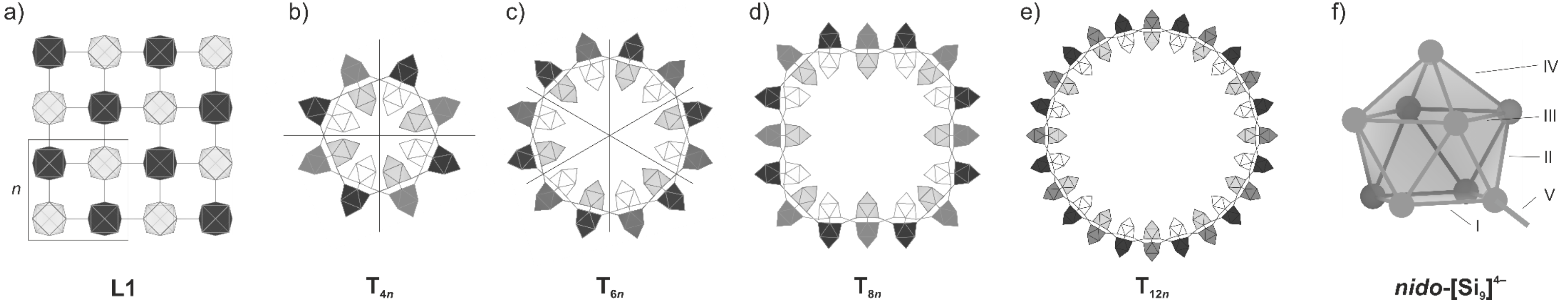

Figure S1. Tubes derived from rolling ***m*** units ***n*** of **L1**. a) 2 × 2 section of **L1**; the unit cell is highlighted with a box. b) $\mathbf{T_{4\mathit{n}}}$ closing after 4 unit cells. c) $\mathbf{T_{6\mathit{n}}}$ closing after 6 unit cells. d) $\mathbf{T_{8\mathit{n}}}$ closing after 8 unit cells. e) $\mathbf{T_{12\mathit{n}}}$ closing after 12 unit cells. f) Building block for the construction of **L1** and of all tubes, *nido*-$[Si_9]^{4-}$. The different bond lengths displayed in Table S1 refer to the labels in this figure.

2. Layer with {Si_9} clusters on top of each other and connected via sp^3-Si linkers

The layer with opposing Si_9 clusters, $\{^2_\infty([Si_9]_2–Si_2)_n\}^{\text{on-top}}$ (**L3**), shows clusters with C_4 symmetry in layer group p422. For this structure, the deformation of the building block *nido*-$[Si_9]^{4–}$ is comparable to that in **L1** and **L2** (main text) with slightly larger distances within the open square (2.38 Å, Table S2). The relative energy of this structure exposing the linking Si atoms to highly strained bond angles (94.2° to 148.2°) is highest in energy of all considered layers with a value of ΔE = 0.05 eV per atom. We thus conclude that the direct connection of Si_9 units is more favorable than the maximal deformation of the bonding orbitals of a sp^3-hybridized Si atom. The (indirect) band gap of **L3** is 0.65 eV per atom respectively (full band structures and density of states below).

Table S2. Structural analysis of **L3** (Figure S2).

**No.**	**bond analysis (compare Figure S1)/Å**					
	I	II	III	IV	V	$d1/d2$
**L3**	2.38	$2.50^{\#}$	2.77	2.51	2.36	1.00

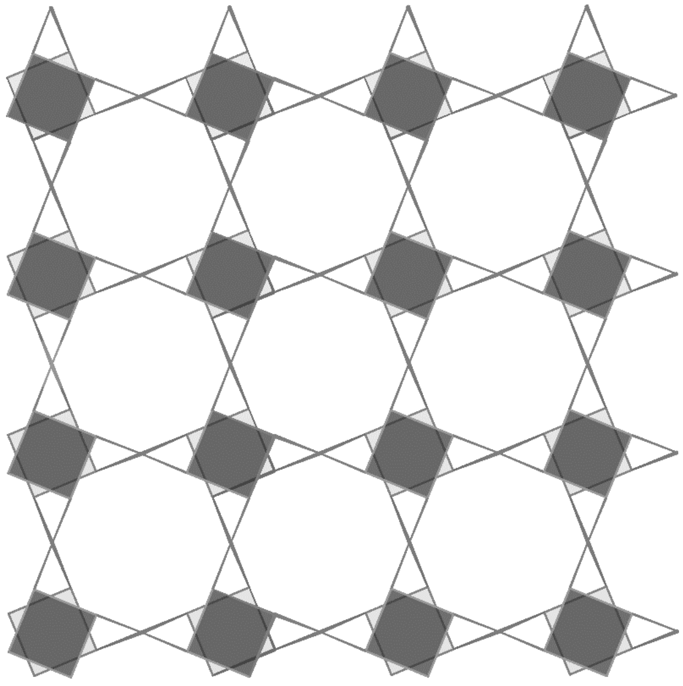

Figure S2. Schematic representation of layer L3. The grey and white squares represent {Si9} clusters above and below the plain, respectively, spanned by the sp^3-hybridized Si atom linkers (grey frame).

3. Population analyses for the basic $[Si_9]^{4-}$ unit in comparison to $[Ge_9]^{4-}$

Table S3. Partial atomic charges from natural population analysis (NPA) and Hirshfeld analysis. The label **a** refers to the open square, **b** to the capped square and **c** to the cap (Figure next to the table). All values are given per atom. The values denoted with * are averaged.

	NPA/e^-		Hirshfeld/e^-	
	**$[Si_9]^{4-}$**	**$[Ge_9]^{4-}$**	**$[Si_9]^{4-}$**	**$[Ge_9]^{4-}$**
a	-0.50*	-0.51	-0.48*	-0.48*
b	-0.36*	-0.35	-0.40	-0.40
c	-0.54	-0.55	-0.48	-0.49

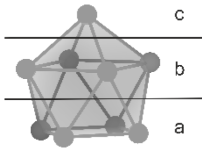

Even though absolute values of the partial charges have no experimentally observable counterparts, the relative values can be considered for an evaluation of the reactions that these clusters may undertake. It can be seen that the open square, which plays a key part in our theoretical investigations, has the higher partial charge than the capped square for both elements Si and Ge.

4. Band structures and density of state (DOS) maps for considered one- and two-dimensional Si modifications

For all calculated two- and one-dimensional structures, we performed band structure and DOS calculations with CRYSTAL.

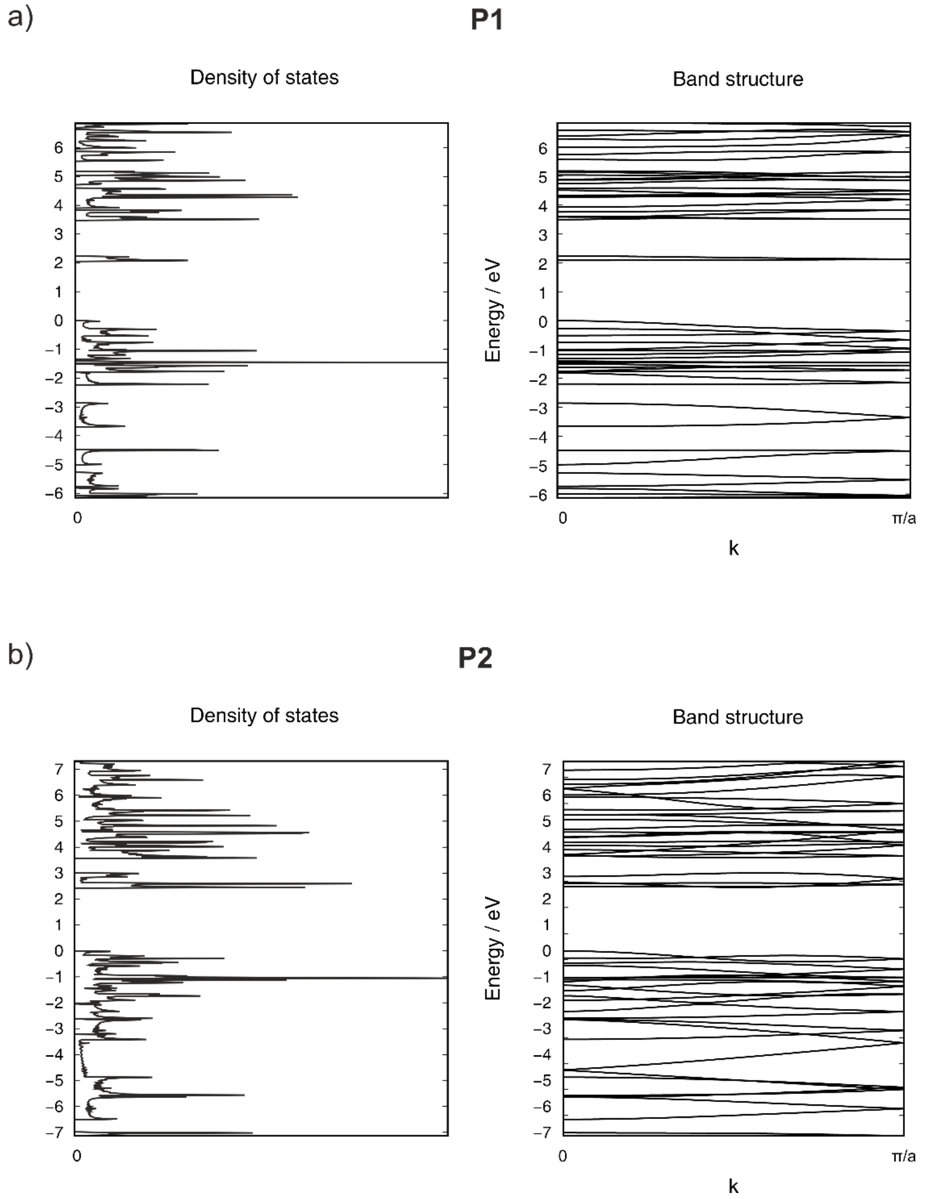

Figure S3. Band Structures and DOSs of polymers P1 and P2.

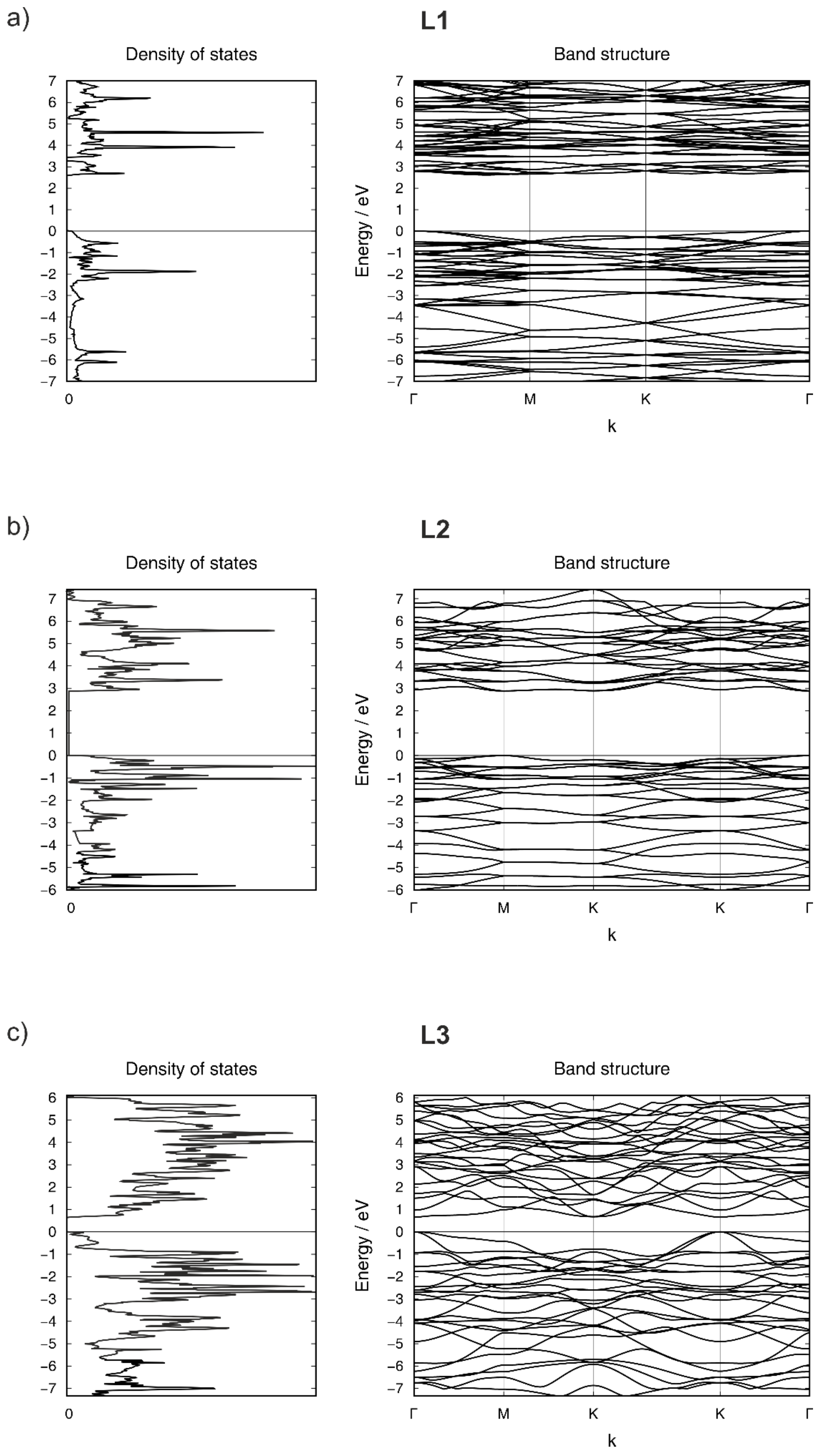

Figure S4. Band Structures and DOSs of layers L1–L3.

5. Structural data of the studied structures

Structural parameters of optimized Si (and Ge) structures given in the CRYSTAL input format (or xyz coordinates for molecular systems studied with Gaussian09 program package).

layer group number	rod group number	point group number
Minimal set of lattice parameters a, b, α, β	Minimal set of lattice parameters a, α	
Number of non-equivalent atoms in the asymmetric unit	Number of non-equivalent atoms in the asymmetric unit	Number of non-equivalent atoms in the asymmetric unit
<atomic number> < fractional x> <fractional y> <z>	<atomic number> <fractional x> <y> <z>	<atomic number> <x> <y> <z>

Silicene

```
72
3.85718051
1
14    -3.333333333333E-01  3.333333333333E-01  2.227497885518E-01
```

Si_{54} nanocluster

```
1
54
14     1.133920491987E+00  9.601811455037E-01  1.686318285429E+00
14    -1.201397891989E-01  3.022237828676E+00  2.710838963430E+00
14     3.902442966502E+00  5.593451656680E-01  5.219939829769E+00
14     2.810690682409E+00 -1.743005350290E-01  3.156938226153E+00
14     2.203872581381E+00  3.783326128353E+00  3.694746144071E+00
14     3.052512422696E+00  2.801446910506E+00 -7.961816263566E-02
14     4.779819926555E+00  4.389543299353E+00  5.713541344359E-01
14     3.836564683787E+00  1.563973696432E+00  1.832922926218E+00
14     6.149142916125E+00  9.378355064987E-01  1.427800044588E+00
14     5.528450162878E+00  2.962967650876E+00  2.702360664252E+00
14     9.268609353221E+00  2.049020535575E+00  3.601688106079E+00
14     7.085077154843E+00  1.101204856044E+00  3.558930879656E+00
14     6.589863803397E+00  4.338261085523E+00  4.320023343228E+00
14     6.796860907767E+00  3.077148108143E+00  5.408410172129E-01
14     8.197774742192E+00  3.788772138552E+00  2.318790042235E+00
14     1.549302600776E+00  3.965093122135E+00  1.320206856654E+00
14     1.510546049627E+00  6.305221015117E+00  1.707418205322E+00
14     2.907906066465E-01  7.124959710983E+00  3.646955355163E+00
14     5.190409143322E+00  6.306653859954E+00  4.070146369973E+00
14     3.721804738968E+00  5.301973952257E+00  2.490213103052E+00
14     2.429344041091E+00  8.167302014973E+00  3.698032973685E+00
14     3.379746958515E+00  7.515845360794E+00  1.073654326341E+00
14     3.496718259516E+00  9.587741943639E+00  2.186950348880E+00
14     5.300525409824E+00  6.651520740601E+00 -4.827254133864E-02
14     6.397331222050E+00  7.123408623259E+00  2.056838945205E+00
14     5.597832909562E+00  8.971755572673E+00  3.187126744049E+00
14     9.725527288633E+00  6.854327976146E+00  4.689445658592E+00
14     8.186653211740E+00  5.945500228021E+00  3.080119019752E+00
14     6.598717407378E+00  1.000501751723E+01  5.004630081596E+00
14     1.399981288895E+00  1.620718294953E+00  4.050541794583E+00
14     7.672935852705E-01  1.413586749025E+00  6.305070217597E+00
14     9.387169143311E-01  3.663553588839E+00  6.907765898912E+00
14     3.344449716260E+00  2.432896293791E+00  8.400760810153E+00
14     2.675537428825E+00  3.659899763797E-01  7.307931374760E+00
14     1.508433863043E+00  3.723802457871E+00  9.279616323909E+00
14     3.032149701397E+00  2.587382333583E+00  6.031956007056E+00
14     4.829115934527E+00  5.089982316496E+00  7.347023783233E+00
14     6.187077976794E+00  6.438719224872E-02  5.462560093872E+00
14     6.592865057869E+00  2.038983213073E+00  6.559528523594E+00
14     5.600755044452E+00  3.136881967828E+00  8.400402727376E+00
14     6.648537224659E+00  4.682514210328E+00  1.006221950713E+01
```

```
14      8.322512278813E+00  3.145670015343E+00  5.506681424780E+00
14      8.889925019056E+00  5.255386124675E+00  6.310820475491E+00
14      1.121382404716E+00  5.284730438784E+00  5.127900746057E+00
14      2.818795364225E+00  6.109978213171E+00  6.529180780539E+00
14      4.893653731718E+00  6.402694817319E+00  9.465795413716E+00
14      2.816403065694E+00  5.652755897841E+00  8.794553374043E+00
14      3.946043371995E+00  7.826758918900E+00  5.475219481122E+00
14      4.700485311591E+00  9.771959869318E+00  6.540845856359E+00
14      6.651921503330E+00  6.379325289724E+00  6.504697571102E+00
14      7.866093102148E+00  8.021626639573E+00  7.976136289886E+00
14      5.550870285272E+00  8.165782655068E+00  8.014780592918E+00
14      8.145692022297E+00  5.763367475257E+00  8.436385846090E+00
14      7.937413148632E+00  8.188816892168E+00  5.551102194679E+00
```

S_2 $(Si_9)_2$

```
9
14      2.360547660136E+00  7.159684385827E-01  2.619558139502E+00
14     -1.808105055005E+00  9.222772046746E-02  3.363657466768E+00
14      1.236153922487E-01  1.928191670430E+00  3.204523586593E+00
14     -7.007504786087E-01 -2.265924154082E+00  2.865888457218E+00
14      1.325535634630E+00 -1.396388541941E+00  1.508021204675E+00
14     -1.572346589613E+00  1.584428891127E+00  1.335668628384E+00
14      8.107441745896E-01 -1.039286918730E+00 -9.136185598023E-01
14     -1.126634800493E+00  8.426561128225E-01 -1.083735331338E+00
14      5.871669751140E-01 -4.361764264969E-01  3.849022757979E+00
```

S_3 $(Si_9)_3$

```
27
14      2.072834698782E+00  1.271018390139E+00  1.538173927387E+00
14     -2.124879210009E+00 -9.988457992205E-01 -1.427748451701E+00
14     -1.632716344062E+00  1.152882402626E+00  1.059505250261E+00
14     -2.314395304023E-01  1.929081045551E+00 -9.001906651906E-01
14      1.025977996214E-01 -2.931635522328E+00  1.249922562293E+00
14      2.427345531548E+00 -1.482682692171E+00 -1.290192182217E+00
14      1.306745892323E-01  2.690050488062E+00  1.417805398633E+00
14     -2.583042100754E+00  1.410966852238E+00 -1.254568574415E+00
14      1.700388242769E+00 -1.076116661497E+00  1.095609681150E+00
14      5.119574843010E-02 -1.978344456669E+00 -1.092478972032E+00
14     -1.519252647231E+00 -1.141669740178E+00  8.736369963584E-01
14      1.763550787193E+00  8.017842737641E-01 -7.883279074686E-01
14      4.080063590644E+00 -6.861357717746E-02  1.991834394835E+00
14     -2.050176041996E+00 -3.391877666268E+00 -2.007710153560E+00
14     -2.071991960703E+00  3.668136149559E+00  1.903918621355E+00
14     -1.496393615428E+00  3.340614354003E+00 -2.348764681209E+00
14     -2.198913432295E+00 -2.933827816536E+00  2.200427956675E+00
14      3.605566394269E+00  5.112267747061E-01 -2.199565893057E+00
14      3.743441780211E+00 -2.181117225006E+00  6.711937326826E-01
14     -7.002936019659E-03 -4.314798121815E+00 -8.003881377720E-01
14     -2.326309678925E-01  4.326606257246E+00 -3.263803709829E-01
14     -3.760881608448E+00  2.118754151976E+00  7.819122291563E-01
14     -3.557321087489E+00 -2.310040996828E+00  2.040203816968E-02
14      3.818903838351E+00  1.947706419887E+00  1.637515282292E-02
14      5.114645835284E+00 -1.438203654179E-01 -2.168972571167E-01
14     -2.412291815741E+00 -4.499676640033E+00  1.515848546427E-01
14     -2.730789110201E+00  4.279836457464E+00 -3.106896494999E-01
```

S_6 $(Si_9)_6$

```
45
3
14      1.392639241185E+00  1.392639241185E+00 -5.310320809098E+00
14      1.676185001385E+00  1.384675647351E-20 -3.301183040813E+00
14     -4.801933974376E-37 -4.119441072941E-37 -6.860235754815E+00
```

S_{12} $(Si_9)_{12}$

```
45
4
14      2.798608639532E+00  4.473491637750E+00 -1.172994781839E+00
14      6.462785476067E+00 -3.719504822795E+00 -3.102546040290E-18
14      1.899609087659E+00  5.082121006295E+00  5.082121006295E+00
14      5.411511272584E-19  6.217496094246E+00  6.217496094246E+00
```

S30 $(Si_9)_{30}$

```
270
14    -3.713155456469E+00 -8.070017309013E+00  1.170912972089E-01
14    -5.690850378240E+00 -6.822016138660E+00 -5.644495515902E-02
14    -3.398843626379E+00 -7.897148020490E+00 -2.233063901418E+00
14    -1.919563550036E+00 -8.547055577943E+00  1.487660580992E+00
14    -5.376668675931E+00 -6.649999792267E+00 -2.406798904273E+00
14    -7.047928427466E+00 -5.311236495025E+00  1.038512425766E+00
14    -1.301324561394E+00 -8.206123545725E+00 -3.143029552823E+00
14    -9.484299646991E-01 -8.080685298101E+00  3.570288912585E+00
14     2.424989368088E-01 -8.864143561304E+00  5.506779757448E-01
14    -6.425460329401E+00 -4.970825229751E+00 -3.591305162192E+00
14    -7.296754545747E+00 -4.073336349199E+00  3.014130451001E+00
14    -8.129829918008E+00 -3.581152118427E+00 -1.821002804205E-01
14     1.707804745689E-01 -7.464497014449E+00 -4.810678907903E+00
14     5.467514927934E-01 -8.698351254806E+00 -1.730426745912E+00
14     1.213592082333E+00 -8.397789926461E+00  2.633558527604E+00
14    -1.196455859052E+00 -6.863752027283E+00  5.514672509077E+00
14    -6.175643035744E+00 -3.459758583575E+00 -5.366905856464E+00
14    -7.823370494066E+00 -3.413619966140E+00 -2.462747963718E+00
14    -8.378259230004E+00 -2.343272282256E+00  1.793962100756E+00
14    -6.340600996132E+00 -3.614536905156E+00  5.063159264207E+00
14     2.018662101448E+00 -7.956683051170E+00 -3.398279232279E+00
14     4.182659215677E-01 -5.977726796045E+00 -6.557509286915E+00
14     3.064076817810E+00 -7.488947985793E+00  3.669428485756E+00
14    -2.573586335371E+00 -5.326635735630E+00  6.627953037400E+00
14     7.104818104600E-01 -5.926328521692E+00  6.581371967801E+00
14    -7.573541681093E+00 -1.902514697705E+00 -4.238246473917E+00
14    -4.547335383771E+00 -4.022770378667E+00 -9.132958326217E+00
14    -8.476268256769E+00 -2.073950245781E-01  2.660730017523E+00
14    -4.520109948643E+00 -4.099547776248E+00  6.458972578117E+00
14    -6.438857999886E+00 -1.413240717398E+00  5.955591954615E+00
14     4.058416835067E+00 -6.945874403103E+00 -3.773024485632E+00
14    -6.488815185034E-01 -4.271423082396E+00 -7.760327232897E+00
14     2.520377349944E+00 -4.936384749130E+00 -6.944439185388E+00
14     5.135831059804E+00 -6.459299124247E+00  3.288800776385E+00
14     2.808888833309E+00 -6.235499554563E+00  5.673670751306E+00
14    -6.667525799007E-01 -4.389902988007E+00  7.694958316635E+00
14    -7.478920593220E+00  3.375839418329E-01 -4.782355368524E+00
14    -2.594730272442E+00 -3.043547864232E+00 -7.930304913832E+00
14    -4.631980383268E+00 -4.224774383406E-01 -7.571427304704E+00
14    -8.376783708663E+00  2.068414167454E+00  2.105221980815E+00
14    -7.491358485317E+00  2.650239412407E-01  4.772825494874E+00
14    -4.617853557349E+00 -1.897749452155E+00  7.351238873445E+00
14     5.939240642097E+00 -6.022123883035E+00 -2.720450584081E+00
14     4.312733862196E+00 -5.412179080227E+00 -5.571772260746E+00
14     1.452785619541E+00 -3.229547409022E+00 -8.147134645995E+00
14     4.880595554387E+00 -5.205223755142E+00  5.292437841260E+00
14     6.584064136144E+00 -5.731564974136E+00  1.646972731192E+00
14    -7.611739784595E-01 -2.252716494672E+00  8.558951882413E+00
14    -7.576470705997E+00  2.509259206002E+00 -3.903460752828E+00
14    -5.987107483119E+00  1.088392405747E+00 -6.474298409155E+00
14    -2.498809269216E+00 -7.358423458917E-01 -8.493188864533E+00
14    -7.391839612144E+00  2.540705510028E+00  4.216582419614E+00
14    -8.132828753106E+00  3.557139105997E+00  3.595851305475E-01
14    -2.766404145525E+00 -9.878659734786E-01  8.384440031613E+00
14     6.193538782753E+00 -4.488761608667E+00 -4.519708702771E+00
14     6.894922712534E+00 -5.563267047703E+00 -6.711329707872E-01
14     1.545889861472E+00 -9.902093366004E-01 -8.695025106036E+00
14     6.083936758266E+00 -3.259990627625E+00  5.595135734392E+00
14     7.820871010233E+00 -3.725442147230E+00  1.958095672508E+00
14     4.595435695784E-01 -2.752117928024E-01  8.869810087746E+00
14    -6.083936730873E+00  3.259990680534E+00 -5.595135720201E+00
14    -7.820871047618E+00  3.725442200457E+00 -1.958095685297E+00
14    -4.595435654910E-01  2.752118065063E-01 -8.869810150618E+00
14    -6.193538741573E+00  4.488761607922E+00  4.519708687740E+00
14    -6.894922727965E+00  5.563267084203E+00  6.711329728361E-01
14    -1.545889857942E+00  9.902093613016E-01  8.695025107585E+00
14     7.391839646584E+00 -2.540705485823E+00 -4.216582452089E+00
14     8.132828745144E+00 -3.557139066125E+00 -3.595851612811E-01
14     2.766404147614E+00  9.878659890939E-01 -8.384440040766E+00
14     5.987107480422E+00 -1.088392352644E+00  6.474298375091E+00
14     7.576470688196E+00 -2.509259153691E+00  3.903460736395E+00
14     2.498809283497E+00  7.358423789845E-01  8.493188771402E+00
14    -4.880595515134E+00  5.205223807343E+00 -5.292437811986E+00
14    -6.584064131849E+00  5.731565007621E+00 -1.646972709561E+00
```

14	7.611740069254E-01	2.252716518361E+00	-8.558951903450E+00
14	-4.312733855124E+00	5.412179122308E+00	5.571772277406E+00
14	-5.939240622850E+00	6.022123898491E+00	2.720450585366E+00
14	-1.452785604620E+00	3.229547446115E+00	8.147134616190E+00
14	7.491358507973E+00	-2.650239306380E-01	-4.772825549261E+00
14	8.376783733472E+00	-2.068414140926E+00	-2.105222012728E+00
14	4.617853553917E+00	1.897749466360E+00	-7.351238889999E+00
14	7.478920615381E+00	-3.375839127443E-01	4.782355346110E+00
14	4.631980386100E+00	4.224774888773E-01	7.571427236817E+00
14	2.594730289513E+00	3.043547914098E+00	7.930304891100E+00
14	-2.808888795325E+00	6.235499578638E+00	-5.673670704219E+00
14	-5.135831020986E+00	6.459299160336E+00	-3.288800731733E+00
14	6.667526060817E-01	4.389903009900E+00	-7.694958320600E+00
14	-4.058416790091E+00	6.945874410446E+00	3.773024458800E+00
14	-2.520377343667E+00	4.936384803327E+00	6.944439196463E+00
14	6.488815461625E-01	4.271423124078E+00	7.760327253139E+00
14	8.476268300384E+00	2.073950507461E-01	-2.660730071819E+00
14	6.438858002007E+00	1.413240714737E+00	-5.955591975419E+00
14	4.520109963210E+00	4.099547779542E+00	-6.458972602121E+00
14	7.573541720023E+00	1.902514720867E+00	4.238246429801E+00
14	4.727951671527E+00	2.730529709371E+00	7.008647350488E+00
14	-3.064076763674E+00	7.488948013817E+00	-3.669428428940E+00
14	-7.104817630907E-01	5.926328531299E+00	-6.581371921854E+00
14	2.573586364680E+00	5.326635745771E+00	-6.627953038161E+00
14	-2.018662029587E+00	7.956683035628E+00	3.398279206744E+00
14	-4.182659113558E-01	5.977726862234E+00	6.557509303897E+00
14	8.378259276063E+00	2.343272300809E+00	-1.793962156275E+00
14	6.340601011139E+00	3.614536890109E+00	-5.063159289197E+00
14	7.823370527899E+00	3.413619966817E+00	2.462747915737E+00
14	6.175643089586E+00	3.459758629119E+00	5.366905807443E+00
14	-1.213592016325E+00	8.397789947455E+00	-2.633558486005E+00
14	1.196455891765E+00	6.863752041458E+00	-5.514672491933E+00
14	-5.467514367166E-01	8.698351247590E+00	1.730426762775E+00
14	-1.707804414659E-01	7.464497070351E+00	4.810678923668E+00
14	8.129829970608E+00	3.581152124128E+00	1.821002384772E-01
14	7.296754560995E+00	4.073336346647E+00	-3.014130490054E+00
14	6.425460386280E+00	4.970825254604E+00	3.591305127183E+00
14	-2.424988779162E-01	8.864143570366E+00	-5.506779464473E-01
14	9.484300117444E-01	8.080685307634E+00	-3.570288889384E+00
14	1.301324617760E+00	8.206123582206E+00	3.143029570908E+00
14	7.047928463233E+00	5.311236519998E+00	-1.038512466390E+00
14	5.376668757869E+00	6.649999831071E+00	2.406798893553E+00
14	1.919563611566E+00	8.547055574345E+00	-1.487660557442E+00
14	3.398843705149E+00	7.897148071861E+00	2.233063903524E+00
14	5.690850411078E+00	6.822016157603E+00	5.644493928301E-02
14	3.713155515746E+00	8.070017308597E+00	-1.170912975412E-01
14	-4.727951634673E+00	-2.730529654441E+00	-7.008647411985E+00
14	6.135310545048E+00	-8.320177772197E+00	-3.667529320922E+00
14	7.178016564840E+00	-2.835537753938E+00	7.795033412466E+00
14	-2.864775776646E+00	4.849256856842E+00	9.411882648886E+00
14	-1.011097994613E+01	4.114589561089E+00	-1.051139014805E+00
14	8.098771264625E+00	-7.376711108982E+00	5.443710922642E-01
14	-2.703281866074E+00	-7.722961747709E+00	7.305124321508E+00
14	-1.061746533022E+01	1.062822682342E+00	2.537046962341E+00
14	-4.708920565110E+00	6.833659420785E+00	-7.172577866934E+00
14	6.860143313039E+00	1.620588916528E+00	-8.404405692456E+00
14	4.391433926112E+00	2.697276390442E-01	1.004795882769E+01
14	-6.701399166472E+00	-5.200360787426E+00	6.953187129760E+00
14	-7.523356598145E+00	-5.520005387934E+00	-5.761169514003E+00
14	3.058109027936E+00	-2.456982194735E-01	-1.053156575495E+01
14	1.042329549158E+01	3.327558108901E+00	-7.609202095151E-01
14	-6.549351705476E+00	5.826559830240E+00	6.592881807353E+00
14	-8.664436482035E+00	-6.140647890894E+00	2.745559173997E+00
14	1.945361778337E+00	-1.039872266285E+01	-2.899881225215E+00
14	1.061746535735E+01	-1.062822678813E+00	-2.537046961415E+00
14	5.367182521777E+00	8.966647354440E+00	3.327996388600E+00
14	-9.604447848376E+00	1.614602235460E+00	-5.044097108008E+00
14	-5.878995370396E+00	-9.246178605840E+00	4.939407231247E-01
14	4.708920609270E+00	-6.833659456663E+00	7.172577876352E+00
14	7.523356695957E+00	5.520005407671E+00	5.761169462261E+00
14	-1.321779843634E+00	1.074041368938E+01	-1.788006634326E+00
14	-5.529458056198E-01	-6.543582587543E+00	-8.782760147267E+00
14	-2.194071718865E+00	-1.022489263837E+01	3.311031791789E+00
14	-3.058109041179E+00	2.456982741857E-01	1.053156575269E+01
14	-1.945361792737E+00	1.039872258976E+01	2.899881268469E+00
14	-3.978048408732E-01	6.199542064179E+00	-9.039587593976E+00
14	9.015874869945E+00	-1.831578459559E+00	-5.970827225983E+00
14	-1.059597027261E+00	-9.598426143413E+00	-5.196816092085E+00

14	-1.042329543206E+01	-3.327558114736E+00	7.609202004928E-01
14	-6.135310477400E+00	8.320177827686E+00	3.667529152003E+00
14	5.878995373666E+00	9.246178639813E+00	-4.939407344100E-01
14	9.796373820489E+00	2.986898536254E+00	3.926724752642E+00
14	5.123273133998E+00	-8.872835899762E+00	3.914221226034E+00
14	-5.367182338054E+00	-8.966647312010E+00	-3.327996392616E+00
14	-7.178016630359E+00	2.835537770029E+00	-7.795033418276E+00
14	2.194071733466E+00	1.022489262687E+01	-3.311031764265E+00
14	1.059597090384E+00	9.598426146678E+00	5.196816117615E+00
14	2.026525309179E-01	-1.811984671249E+00	1.081596106522E+01
14	1.321779947594E+00	-1.074041362975E+01	1.788006571544E+00
14	2.864775791807E+00	-4.849256760798E+00	-9.411882621677E+00
14	2.703281853222E+00	7.722961814862E+00	-7.305124329033E+00
14	-5.123273094288E+00	8.872835917383E+00	-3.914221089722E+00
14	-9.015874915265E+00	1.831578440758E+00	5.970827106171E+00
14	3.978048927983E-01	-6.199542023814E+00	9.039587653314E+00
14	1.011097993120E+01	-4.114589532682E+00	1.051139005884E+00
14	6.701399183819E+00	5.200360746810E+00	-6.953187179768E+00
14	-2.026524511939E-01	1.811984624882E+00	-1.081596117806E+01
14	-9.796373786810E+00	-2.986898550743E+00	-3.926724886839E+00
14	-6.860143319717E+00	-1.620588767829E+00	8.404405642732E+00
14	4.547335415382E+00	4.022770430975E+00	9.132958256667E+00
14	8.664436368409E+00	6.140647900375E+00	-2.745559352921E+00
14	9.604447857116E+00	-1.614602208885E+00	5.044097045902E+00
14	6.549351706879E+00	-5.826559868454E+00	-6.592881798151E+00
14	-4.391433961890E+00	-2.697275290628E-01	-1.004795888711E+01
14	-8.098771231370E+00	7.376711187163E+00	-5.443710761238E-01
14	5.529457614071E-01	6.543582674604E+00	8.782760175103E+00
14	8.860896266610E+00	-5.879081104621E+00	2.629762428114E+00
14	2.000170662131E+00	-7.045162310923E+00	-8.148045973452E+00
14	-8.309961398866E+00	5.082333257478E-01	-7.110289576293E+00
14	-7.824955735245E+00	6.339886295106E+00	4.295149859065E+00
14	2.784266748601E+00	2.393895468385E+00	1.032024213981E+01
14	-2.784266691159E+00	-2.393894054926E+00	-1.032024264261E+01
14	-9.341959899352E+00	-5.709252351627E+00	1.910134606037E-01
14	-2.391331772063E+00	-2.345219174428E+00	1.042244880888E+01
14	8.463145941485E+00	3.060595789239E+00	6.247602566921E+00
14	8.215650123445E+00	3.025613855066E+00	-6.571701676211E+00
14	-8.860896064438E+00	5.879081295442E+00	-2.629762412944E+00
14	-8.215650152885E+00	-3.025613869987E+00	6.571701607160E+00
14	3.279672751811E+00	-8.668295418586E+00	5.845788877450E+00
14	9.732823141684E+00	-3.244281206327E+00	-3.812614299459E+00
14	2.241771041796E+00	5.747884504309E+00	-9.053451685575E+00
14	-2.000170558668E+00	7.045161975287E+00	8.148045621346E+00
14	-2.241772343857E+00	-5.747885381622E+00	9.053451260044E+00
14	-7.200468044723E-01	-1.058638801961E+01	-2.731269406371E+00
14	4.623468795540E-01	-7.790231988468E-01	-1.092090447069E+01
14	-3.310911936668E-01	1.010828457545E+01	-4.193621398533E+00
14	8.309961385839E+00	-5.082332698299E-01	7.110289579738E+00
14	3.310912994268E-01	-1.010828453452E+01	4.193621420818E+00
14	-8.852483536038E+00	-5.444715265950E+00	-3.438402437977E+00
14	-6.549906448818E+00	7.035346132076E+00	-5.239244153266E+00
14	4.057234033622E+00	1.008688397471E+01	1.279348753361E+00
14	7.824955824461E+00	-6.339886245999E+00	-4.295149815761E+00
14	-4.057233828590E+00	-1.008688391098E+01	-1.279348881040E+00
14	-9.895770403851E+00	-3.510723020690E-01	4.694382517441E+00
14	-1.605712507218E+00	9.406980636485E+00	5.363577238725E+00
14	9.341959972437E+00	5.709252359046E+00	-1.910134342300E-01
14	2.391331742116E+00	2.345219113421E+00	-1.042244885152E+01
14	1.605712545944E+00	-9.406980574399E+00	-5.363577322859E+00
14	-6.421523793880E-02	-8.171258029543E+00	7.288137602398E+00
14	-3.119512701537E-01	4.345228616563E+00	1.004234958130E+01
14	1.202721194146E+00	1.085314146831E+01	-9.037740621474E-01
14	-3.279672464767E+00	8.668295311716E+00	-5.845789002959E+00
14	-8.463146256893E+00	-3.060595747923E+00	-6.247602552230E+00
14	-1.202720741428E+00	-1.085314172769E+01	9.037739913200E-01
14	8.469173406411E+00	-3.941949748380E+00	5.725796831794E+00
14	7.186447312536E+00	8.122156647057E+00	1.555118888614E+00
14	7.200464024018E-01	1.058638803542E+01	2.731268835403E+00
14	-9.732823079509E+00	3.244281263764E+00	3.812614270257E+00
14	-7.186447695185E+00	-8.122157360753E+00	-1.555118664943E+00
14	4.857556306851E+00	-7.803465529697E+00	-5.963778344932E+00
14	9.742644058709E+00	3.756728665905E+00	-3.314654835013E+00
14	8.852483584392E+00	5.444715237201E+00	3.438402398243E+00
14	-4.623475770516E-01	7.790235117759E-01	1.092090471435E+01
14	-9.742643466552E+00	-3.756729096320E+00	3.314655252597E+00
14	-6.152112410636E+00	-1.897770581734E+00	-8.856563894435E+00
14	5.343246857584E+00	3.795101614026E+00	-8.782532433390E+00

```
14     9.895770297028E+00  3.510726200252E-01 -4.694381576531E+00
14     6.549906543293E+00 -7.035346010039E+00  5.239244188621E+00
14    -5.343247266021E+00 -3.795102089795E+00  8.782532824269E+00
14    -9.345613761428E+00  5.609237722522E+00  1.033870538923E+00
14     6.421524496867E-02  8.171258020755E+00 -7.288137580008E+00
14    -8.469173391478E+00  3.941949698170E+00 -5.725796571780E+00
14     3.119512531865E-01 -4.345228737608E+00 -1.004234957183E+01
14     9.345613704548E+00 -5.609237736442E+00 -1.033870575983E+00
14     6.152112434030E+00  1.897770613074E+00  8.856563835502E+00
14    -4.857556238303E+00  7.803464808382E+00  5.963779212038E+00
14     9.647086921613E+00 -2.557869070653E+00  7.387752233650E+00
14    -1.322376127263E+00  6.551270658929E+00  1.046349816120E+01
14    -1.047504101755E+01  6.602474104063E+00 -9.162314340576E-01
14    -5.141982562766E+00 -2.474003887681E+00 -1.102741171169E+01
14     7.296680533414E+00 -8.136771745998E+00 -5.894420943047E+00
14    -1.129182004843E+01  1.668295456604E+00  4.886268369790E+00
14    -5.658206341160E+00  9.033479443841E+00 -6.369395772588E+00
14     7.802185597189E+00  3.916776518375E+00 -8.830414120120E+00
14     1.047504100213E+01 -6.602474120042E+00  9.162313655211E-01
14    -1.328842922738E+00 -8.009564322999E+00  9.395259528924E+00
14    -9.962621477100E+00 -4.891196424475E+00 -5.565979346759E+00
14     1.648705524139E+00  9.013668014695E-01 -1.227457878417E+01
14     1.097905643257E+01  5.441200438008E+00 -2.011220581565E+00
14     5.141982539770E+00  2.474003893005E+00  1.102741162980E+01
14    -7.802186058688E+00 -3.916776199057E+00  8.830414277257E+00
14     5.155127019550E-01 -1.149908985879E+01 -4.655959391649E+00
14     1.129181973583E+01 -1.668295187419E+00 -4.886268650519E+00
14     6.464625334458E+00  1.047553071669E+01  1.624134907928E+00
14    -7.296680354473E+00  8.136771854787E+00  5.894420906608E+00
14    -1.097905619348E+01 -5.441200439431E+00  2.011220764903E+00
14     5.658206600879E+00 -9.033479368285E+00  6.369395801900E+00
14     9.962621330836E+00  4.891196356951E+00  5.565979397420E+00
14     5.050425122317E-01  1.205450625394E+01 -2.937167168748E+00
14    -9.647087015915E+00  2.557869106859E+00 -7.387751965875E+00
14    -6.464625107350E+00 -1.047553124107E+01 -1.624134626939E+00
14    -1.648705560436E+00 -9.013670490146E-01  1.227457888804E+01
14    -5.155125893624E-01  1.149908986040E+01  4.655959198328E+00
14     1.328842667739E+00  8.009563927763E+00 -9.395259632926E+00
14     1.322375488561E+00 -6.551271053203E+00 -1.046349829090E+01
14    -5.050426718349E-01 -1.205450627142E+01  2.937167455558E+00
```

P1 $\{^1_\infty([Si_9]–[Si_9])_n\}$

One imaginary frequency with a value of 98i cm^{-1}, which could be identified as an asymmetric rotation of the clusters along the translational axis.

```
1
10.79713964 90.000000
18
14     2.484969462430E-01 -4.000998093300E-02 -1.801971138589E+00
14    -2.467778270744E-01 -1.230326854390E-01  1.931874529975E+00
14    -2.518907564107E-01  3.608879195400E-02 -1.803234363212E+00
14     2.530712151211E-01  1.193269643240E-01  1.932558891712E+00
14     1.308765508248E-01  2.142132980498E+00 -1.655471232073E+00
14    -1.323289530740E-01 -2.304065507786E+00  1.587712945631E+00
14    -1.503696297987E-01 -2.263503098871E+00 -9.862782608330E-01
14     1.487338852284E-01  2.321142811865E+00  9.062946393000E-01
14     3.498503187560E-01  2.262648165820E+00 -9.875396454710E-01
14    -3.512240127558E-01 -2.328104679652E+00  9.108180768500E-01
14    -3.693745236023E-01 -2.148146691018E+00 -1.651476954077E+00
14     3.674104653515E-01  2.302057510228E+00  1.588697537389E+00
14    -1.056407263281E-01 -1.716886715720E-01  1.150738713810E-01
14     1.067833783511E-01  1.339305282230E-01  1.642294111000E-03
14    -3.932699097476E-01 -1.416120250950E-01  3.017907334000E-03
14     3.944487814299E-01  1.720750748560E-01  1.182388557910E-01
14     2.492260411398E-01  4.123779431345E+00 -9.148622180700E-02
14    -2.507436184496E-01 -4.127407056649E+00 -9.556634157800E-02
```

P2 $\{^1_\infty([Si_9]=[Si_9])_n\}$

```
2
8.31743783
9
14    -2.507395243582E-01 -2.016266542230E+00 -2.774795582859E+00
14    -4.761811865597E-01  2.469619641461E-02 -2.693437367799E+00
14    -2.497963802906E-01  2.067969037699E+00 -2.738689470243E+00
```

```
14    -2.502990866160E-01  3.924312791909E-02 -4.295853454734E+00
14    -1.092950015599E-01  1.213422390424E+00 -7.129782877289E-01
14    -1.088389048115E-01 -1.207735596833E+00 -7.373895817072E-01
14    -3.907493603119E-01  1.223044476775E+00 -7.127461698572E-01
14    -2.392600808835E-02  2.327012368175E-02 -2.698060983564E+00
14    -3.903125480975E-01 -1.197273948611E+00 -7.292872574564E-01
```

L1 $\{{}^{2}_{\infty}[\mathrm{Si}_9]_n\}$-sheet

```
57
10.98548258
6
14    -3.706213871906E-01  1.292871400050E-01  2.430154776020E+00
14    -1.293041547016E-01  1.293041547016E-01  2.429200052533E+00
14    -3.706350604866E-01  3.706350604866E-01  2.431150259183E+00
14    -4.010764899744E-01  2.500205957782E-01  3.716651516473E-01
14    -2.500216027438E-01  9.896987487642E-02  3.701942245505E-01
14    -2.499373007512E-01  2.499373007512E-01  4.049986228889E+00
```

L2 $\left\{{}^{2}_{\infty}([\mathrm{Si}_9]_2\text{–}\mathrm{Si}_2)_{\mathrm{n}}\right\}^{\mathrm{chess}}$

```
1
7.31766944 7.31951448 90.001114
20
14     4.916422488147E-01 -2.618784676376E-01  1.217384735171E+00
14    -2.805562003437E-01 -4.896555304942E-01  1.216933152305E+00
14     4.918449338172E-01  2.823761318017E-01  1.226521746750E+00
14     2.640218120969E-01 -4.898490510557E-01  1.227154287920E+00
14     3.058557135031E-01 -3.028104688143E-01  3.261344124387E+00
14    -3.213354693916E-01 -3.026360585787E-01  3.254059650664E+00
14    -3.210250064397E-01  3.246216709230E-01  3.260520984165E+00
14     3.060872270716E-01  3.243059260203E-01  3.267974531936E+00
14     4.928814869971E-01 -4.886756019349E-01  4.849432298013E+00
14    -7.611145656545E-03 -2.170703290330E-01 -1.186176627696E+00
14     2.200042547047E-01  1.091505540178E-02 -1.196125456333E+00
14    -7.830375685800E-03  2.386989416043E-01 -1.194636313080E+00
14    -2.353919235167E-01  1.071158106517E-02 -1.184175974516E+00
14    -1.947801476447E-01 -1.762079465341E-01 -3.221840782701E+00
14     1.781059020112E-01 -1.760072025093E-01 -3.230265048019E+00
14     1.778863947923E-01  1.968151622322E-01 -3.236566699383E+00
14    -1.951407391801E-01  1.965416864802E-01 -3.227733139412E+00
14    -9.073425075884E-03  9.878142393523E-03 -4.817817627675E+00
14    -7.822550044266E-03 -4.895412350601E-01  1.650125077539E-02
14     4.917982091703E-01  1.063471372963E-02  1.501090673167E-02
```

L3 $\left\{{}^{2}_{\infty}([\mathrm{Si}_9]_2\text{–}\mathrm{Si}_2)_{\mathrm{n}}\right\}^{\text{on-top}}$

```
1
6.31418985 6.31331903 90.014298
20
14    -1.102377586729E-01  2.562997818993E-01  1.620155910384E+00
14     2.381951989554E-01  1.128539060485E-01  1.621305411341E+00
14     9.427531875691E-02 -2.355100071909E-01  1.623509040920E+00
14    -2.542032496900E-01 -9.207912757382E-02  1.622865993783E+00
14    -2.916541787268E-01  1.362937366917E-01  3.673270370178E+00
14     1.176065489620E-01  2.943455023123E-01  3.673460392717E+00
14     2.759289147302E-01 -1.148685670845E-01  3.674693678742E+00
14    -1.335832719729E-01 -2.733854232281E-01  3.674909203809E+00
14    -7.998154950997E-03  1.057480024185E-02  5.235381605502E+00
14    -1.106359613573E-01 -2.355680024838E-01 -1.591605923025E+00
14     2.377290606932E-01 -9.176651297259E-02 -1.589249982748E+00
14     9.425233757362E-02  2.565949643716E-01 -1.588858207119E+00
14    -2.540593520487E-01  1.128702640556E-01 -1.591093568876E+00
14    -2.919508266559E-01 -1.147755766028E-01 -3.643033789009E+00
14     1.175011021132E-01 -2.731775669078E-01 -3.642246881836E+00
14     2.757001631679E-01  1.361406413352E-01 -3.641203462852E+00
14    -1.333056708709E-01  2.945184040877E-01 -3.642152786596E+00
14    -8.013729036660E-03  1.065151767043E-02 -5.204010778465E+00
14    -8.129292893607E-03 -4.891716498968E-01  1.592949873972E-02
14     4.921480019247E-01  1.032453522722E-02  1.547427441224E-02
```

T$_{4n}$ $\{{}^{1}_{\infty}[\mathrm{Si}_9]_{4n}\}$

```
43
11.12935302
24
14    -4.195731670576E-01  9.221210950143E+00  1.356929448706E+00
14     3.248897918819E-01  5.560916446768E+00 -7.480042362284E+00
14     3.225533179322E-01  4.284725135269E+00  1.325520023533E+00
14    -4.171484940954E-01  2.092583773650E+00 -3.967182823146E+00
14     8.062677529980E-02  7.483017270732E+00 -5.563440748818E+00
14    -1.752942601426E-01  9.224795239552E+00 -1.357112653461E+00
14     8.276107240730E-02  4.285909710375E+00 -1.325805254290E+00
14    -1.773672878478E-01  3.968995616556E+00 -2.094109889252E+00
14    -2.971959416447E-01  7.203402450841E+00  1.703896532018E+00
14     2.025021697419E-01  3.888852395002E+00 -6.298513074451E+00
14     2.027567266778E-01  6.419886763474E+00  1.585813707674E+00
14    -2.974591911617E-01  3.418903890427E+00 -5.661477071824E+00
14     5.579246143621E-02  5.055509355921E+00 -5.055509355921E+00
14    -1.504922966838E-01  7.148694975428E+00  0.000000000000E+00
14     4.520553696844E-02  6.349016186384E+00  0.000000000000E+00
14    -1.399005157998E-01  4.490415417003E+00 -4.490415417003E+00
14    -4.438680383370E-01  7.144554617557E+00  0.000000000000E+00
14     3.491766939885E-01  5.052099627894E+00 -5.052099627894E+00
14     3.603536587093E-01  6.347503365364E+00  0.000000000000E+00
14    -4.550533340569E-01  4.488270380398E+00 -4.488270380398E+00
14    -2.976218728834E-01  1.082564462770E+01  0.000000000000E+00
14     2.029457012751E-01  7.654943931586E+00 -7.654943931586E+00
14     2.025610786959E-01  2.646798586397E+00  0.000000000000E+00
14    -2.971020972948E-01  1.872065148282E+00 -1.872065148282E+00
```

T6*n* $\{^{1}_{\infty}[\mathbf{Si}_9]_{6n}\}$

```
88
11.15376508
24
14     8.012248850371E-02  7.578957728856E+00  1.043371604703E+01
14    -1.783390870226E-01 -1.346704043513E+00  1.282398978272E+01
14    -1.805207772034E-01  5.137607372631E+00  6.236386222766E+00
14     8.234615130122E-02 -1.331121607217E+00  7.968672212526E+00
14    -4.198906221330E-01  1.346652880527E+00  1.282486150119E+01
14     3.216794001286E-01  5.245643014142E+00  1.177915393151E+01
14    -4.176548631593E-01  2.831417695887E+00  7.566448699912E+00
14     3.194567897589E-01  1.331083338252E+00  7.969760071905E+00
14     2.008063778550E-01  6.856265425250E+00  8.488042942070E+00
14    -2.991709795173E-01 -1.693618424021E+00  1.077877618397E+01
14    -2.991855608350E-01  6.440114680191E+00  7.923523816593E+00
14     2.008180218954E-01 -1.615599575089E+00  1.008224891398E+01
14    -4.467972026937E-01  2.642273446417E-20  1.074779957925E+01
14     3.484258099944E-01  5.373251440998E+00  9.306744497651E+00
14    -4.546747337868E-01  5.006995411104E+00  8.672370445296E+00
14     3.563052083390E-01  2.492151651875E-20  1.001552030176E+01
14     5.318164614246E-02  5.374217958559E+00  9.308418555173E+00
14    -1.515494622645E-01  2.642160791202E-20  1.074671141546E+01
14    -1.436928550937E-01  5.007711015921E+00  8.673609909197E+00
14     4.534012842004E-02  2.491801730590E-20  1.001408579246E+01
14     2.009772193601E-01  7.220897710820E+00  1.250696171140E+01
14    -2.990631109509E-01  3.392369177770E-20  1.444165909275E+01
14    -2.990172720019E-01  3.164401081135E+00  5.480903448051E+00
14     2.009559453908E-01  1.742863592628E-20  6.328790025315E+00
```

T8*n* $\{^{1}_{\infty}[\mathrm{Si}_9]_{8n}\}$

```
43
11.07849622
42
14    -4.291671164472E-01  1.483801381946E+01 -4.204656852881E+00
14     3.149680281334E-01  1.107389065736E+01 -1.298622510397E+01
14     3.130152743053E-01  9.778227199979E+00 -4.259095699392E+00
14    -4.289312371928E-01  7.548611005543E+00 -9.412570371385E+00
14     7.034083138017E-02  1.298805296027E+01 -1.107545982092E+01
14    -1.849476261209E-01  1.470795887344E+01 -6.895973465240E+00
14     7.209693861307E-02  9.996941548628E+00 -6.916305565809E+00
14    -1.857136851725E-01  9.413684134276E+00 -7.549648089978E+00
14    -4.293206730077E-01  1.471160812251E+01 -6.897237575709E+00
14     3.133790782322E-01  9.999581430514E+00 -6.917264375465E+00
14    -1.852585375677E-01  1.483481706679E+01 -4.203271040304E+00
14     7.269310628981E-02  9.775414555676E+00 -4.258108948972E+00
14    -3.074531606699E-01  1.279971774465E+01 -3.771859051637E+00
14     1.924926206465E-01  9.392283129652E+00 -1.179614405683E+01
```

```
14     1.926716707910E-01  1.184910418557E+01 -3.877806889200E+00
14    -3.074905700672E-01  8.844601130455E+00 -1.111192347210E+01
14     4.469573551681E-02  1.056567659599E+01 -1.056567659599E+01
14    -1.589570858066E-01  1.269624536450E+01 -5.439517196704E+00
14     3.793971278088E-02  1.194319442136E+01 -5.518502925492E+00
14    -1.506410678531E-01  9.964025826797E+00 -9.964025826797E+00
14    -4.558963033305E-01  1.270115169377E+01 -5.440654018589E+00
14     3.402857552995E-01  1.056353761529E+01 -1.056353761529E+01
14     3.472595037925E-01  1.194668673613E+01 -5.520123508469E+00
14    -4.643253167870E-01  9.962673803508E+00 -9.962673803508E+00
14    -3.073855041195E-01  1.266536075689E+01 -7.135890154047E+00
14     1.925085106557E-01  1.217620485098E+01 -7.111938497049E+00
14    -3.070029089744E-01  1.638280579579E+01 -5.635907544042E+00
14     1.927659258072E-01  1.316975328791E+01 -1.316975328791E+01
14     1.929408264294E-01  8.266974935693E+00 -5.720664400865E+00
14    -3.072233842135E-01  7.330376325365E+00 -7.330376325365E+00
14     3.135685510795E-01 -1.337810212734E+00 -1.468058669347E+01
14    -4.278641568631E-01 -1.345258897598E+00 -9.863153981222E+00
14     7.184796729421E-02  1.337819152794E+00 -1.468205963127E+01
14    -1.866943215176E-01  1.345153000902E+00 -9.865346747228E+00
14     1.925998506705E-01 -1.688777581421E+00 -1.261430896531E+01
14    -3.074323813307E-01 -1.648625806819E+00 -1.194013771593E+01
14     4.302074281136E-02  0.000000000000E+00 -1.260997212001E+01
14    -1.558839939514E-01  0.000000000000E+00 -1.193118939137E+01
14     3.421622049740E-01  0.000000000000E+00 -1.260825938381E+01
14    -4.589780223977E-01  0.000000000000E+00 -1.192815574503E+01
14     1.927903881651E-01  0.000000000000E+00 -1.630907101751E+01
14    -3.071773731821E-01  0.000000000000E+00 -8.241376322738E+00
```

T_{12n} $\{^{1}_{\infty}[Si_9]_{12n}\}$

```
88
11.06641875
42
14    -4.288072819074E-01 -2.230818273382E+01 -7.347264974361E+00
14     3.141535445053E-01 -2.340506529675E+01  1.342409317553E+00
14     3.129601840345E-01 -1.758261746808E+01 -6.126178713597E+00
14    -4.276316522293E-01 -1.852302978231E+01  1.336743560808E+00
14     7.119043406076E-02 -2.340551449144E+01 -1.342423062802E+00
14    -1.857991347385E-01 -2.297509779305E+01 -4.746299106277E+00
14     7.240155588902E-02 -1.824603629240E+01 -3.537077197343E+00
14    -1.870165200223E-01 -1.852349540226E+01 -1.336715437992E+00
14    -4.288005672407E-01 -2.297599340958E+01 -4.746354797067E+00
14     3.129897015793E-01 -1.824685665832E+01 -3.537319330690E+00
14    -1.858037661333E-01 -2.230737129409E+01 -7.347215428501E+00
14     7.244243700394E-02 -1.758177654888E+01 -6.126002420088E+00
14    -3.073618017645E-01 -2.023153799962E+01 -7.165995520949E+00
14     1.926426103740E-01 -2.134851498632E+01  1.682584852655E+00
14     1.926410420919E-01 -1.953027139054E+01 -6.945859102214E+00
14    -3.073583881554E-01 -2.061293392647E+01  1.645426141331E+00
14     4.317729342213E-02 -2.132937999458E+01  1.639728216645E-19
14    -1.579724468954E-01 -2.063009131605E+01 -5.530156698715E+00
14     3.926544785022E-02 -1.990545645259E+01 -5.344287757349E+00
14    -1.540603090605E-01 -2.058070234477E+01  1.724980633927E-19
14    -4.567476433420E-01 -2.063116309902E+01 -5.530323523491E+00
14     3.421065446280E-01 -2.132879653025E+01  1.639921006185E-19
14     3.460134992836E-01 -1.990655420194E+01 -5.344524501888E+00
14    -4.606561869577E-01 -2.058007898007E+01  1.725301505705E-19
14    -3.073536849170E-01 -2.106833624262E+01 -3.905186967779E+00
14     1.926380003371E-01 -2.034704046393E+01 -3.759192517339E+00
14    -3.072593405522E-01 -2.421114941139E+01 -6.450088387726E+00
14     1.926936291533E-01 -2.502609461457E+01  1.251800315353E-19
14     1.927427895847E-01 -1.633332311211E+01 -4.425720177891E+00
14    -3.073013121950E-01 -1.689136172534E+01  2.114091435496E-19
14     3.142607749676E-01 -1.964974491567E+01  1.289533847955E+01
14    -4.275134279321E-01 -1.542611234500E+01  1.044909777417E+01
14     7.121449955636E-02 -2.099382684828E+01  1.057025183971E+01
14    -1.870194789229E-01 -1.676351420459E+01  8.135643737131E+00
14     1.926389988875E-01 -1.769954641408E+01  1.216316632244E+01
14    -3.073662152952E-01 -1.708352681546E+01  1.176170950627E+01
14     4.331668697435E-02 -1.852437160060E+01  1.069505093018E+01
14    -1.539056647100E-01 -1.787628148988E+01  1.032087593029E+01
14     3.419545241657E-01 -1.852271807449E+01  1.069409626643E+01
14    -4.608258333718E-01 -1.787457566396E+01  1.031989107124E+01
14     1.928114003971E-01 -2.172491709650E+01  1.254288673379E+01
14    -3.071871123605E-01 -1.468095071580E+01  8.476050847727E+00
```

$[Si_9]^{4–}$ calculated at the PBE0[4]/def2-TZVP/PCM level of theory with the Gaussian09 program package.

```
Si   -0.00013   -0.00004    2.28609
Si   -0.02223    1.85979    0.71668
Si   -1.21581    1.20414   -1.30594
Si   -1.86219    0.00997    0.71922
Si   -1.24724   -1.25248   -1.26819
Si    1.20929   -1.20741   -1.30721
Si    1.24088    1.24928   -1.27162
Si    1.85910   -0.01149    0.71586
Si    0.01929   -1.86129    0.71832
```

$[Ge_9]^{4–}$ calculated at the PBE0[4]/def2-TZVP/PCM level of theory with the Gaussian09 program package.

```
Ge   -0.02562    1.99866    0.76355
Ge   -1.98755    0.01916    0.76223
Ge    2.01007   -0.01937    0.77684
Ge    0.04959   -1.99781    0.77575
Ge    1.34199    1.32981   -1.32835
Ge   -1.26468    1.26584   -1.39508
Ge    1.30086   -1.27585   -1.37926
Ge   -1.30478   -1.34100   -1.32986
Ge    0.00499    0.00560    2.40771
```
